# Evaluation of parameters affecting switchgrass tissue culture: toward a consolidated procedure for *Agrobacterium*-mediated transformation of switchgrass (*Panicum virgatum*)

**DOI:** 10.1186/s13007-017-0263-6

**Published:** 2017-12-19

**Authors:** Chien-Yuan Lin, Bryon S. Donohoe, Neha Ahuja, Deborah M. Garrity, Rongda Qu, Melvin P. Tucker, Michael E. Himmel, Hui Wei

**Affiliations:** 1 Biosciences Center, National Renewable Energy Laboratory, Golden, CO 80401 USA; 2 Department of Biology, Colorado State University, Fort Collins, CO 80523 USA; 3 Department of Crop and Soil Sciences, North Carolina State University, Raleigh, NC 27695-7287 USA; 4 National Bioenergy Center, National Renewable Energy Laboratory, Golden, CO USA

**Keywords:** Switchgrass, Bioenergy crop, Red fluorescent protein (RFP), β-Glucuronidase (GUS) staining, *Agrobacterium*-mediated transformation

## Abstract

**Background:**

Switchgrass (*Panicum virgatum*), a robust perennial C4-type grass, has been evaluated and designated as a model bioenergy crop by the U.S. DOE and USDA. Conventional breeding of switchgrass biomass is difficult because it displays self-incompatible hindrance. Therefore, direct genetic modifications of switchgrass have been considered the more effective approach to tailor switchgrass with traits of interest. Successful transformations have demonstrated increased biomass yields, reduction in the recalcitrance of cell walls and enhanced saccharification efficiency. Several tissue culture protocols have been previously described to produce transgenic switchgrass lines using different nutrient-based media, co-cultivation approaches, and antibiotic strengths for selection.

**Results:**

After evaluating the published protocols, we consolidated these approaches and optimized the process to develop a more efficient protocol for producing transgenic switchgrass. First, seed sterilization was optimized, which led to a 20% increase in yield of induced calluses. Second, we have selected a N_6_ macronutrient/B_5_ micronutrient (NB)-based medium for callus induction from mature seeds of the Alamo cultivar, and chose a Murashige and Skoog-based medium to regenerate both Type I and Type II calluses. Third, *Agrobacterium*-mediated transformation was adopted that resulted in 50–100% positive regenerated transformants after three rounds (2 weeks/round) of selection with antibiotic. Genomic DNA PCR, RT-PCR, Southern blot, visualization of the red fluorescent protein and histochemical β-glucuronidase (GUS) staining were conducted to confirm the positive switchgrass transformants. The optimized methods developed here provide an improved strategy to promote the production and selection of callus and generation of transgenic switchgrass lines.

**Conclusion:**

The process for switchgrass transformation has been evaluated and consolidated to devise an improved approach for transgenic switchgrass production. With the optimization of seed sterilization, callus induction, and regeneration steps, a reliable and effective protocol is established to facilitate switchgrass engineering.

**Electronic supplementary material:**

The online version of this article (10.1186/s13007-017-0263-6) contains supplementary material, which is available to authorized users.

## Background

Switchgrass (*Panicum virgatum*) is indigenous to central North America [1] and has been identified as one of the 1745 C4-type grasses on the planet able to grow in both cool and temperate/warm-season environments [2]. In addition, as a perennial grass with a deep root system, switchgrass is known to provide excellent soil conservation and is compatible with conventional farming practices [3–7]. In general, there are two ecotypes of switchgrass varieties, lowland and upland. Lowland types, such as the cultivars Alamo, Kanlow and Timer, grow in the southern U.S. and generally result in taller, more robust growing plants displaying a bunching habit, later maturation, larger leaves, and coarser stems. Upland types, such as Blackwell, Carthage, Cave-In-Rock (CIR), Pathfinder, Trailblazer, Dacotah, Shawnee and Caddo, grow in the northern U.S. and have different morphological characteristics from the lowland types [8, 9]. Switchgrass is known to have the basic chromosome number (x = 9). Lowland types are identified as tetraploid (2n = 4x = 36 chromosomes) and, in rare instances, found as octoploid (2n = 8x = 72 chromosomes). In contrast, upland types are both tetraploid and octoploid, with octoploid being more predominant [10].

Switchgrass possesses many agronomic advantages over C3-type plants and other grass species including: pest and disease tolerance, water use efficiency (switchgrass is two times more efficient than traditional cool season grasses), low fertilizer requirements, and lower harvesting costs [11]. Switchgrass has been evaluated as and is being developed as a dedicated energy crop by a 10-year U.S. DOE-sponsored program [12, 13]; as well as a USDA-sponsored program [14]. The annual biomass yield (dry mass) of switchgrass in the U.S. has been estimated at 12.9 ± 5.9 and 8.7 ± 4.2 Mg ha^−1^ for lowland and upland types, respectively [15]. In addition, switchgrass has been proposed as a dual purpose crop for both forage and bioenergy uses [16].

Because switchgrass has a high degree of self-incompatibility and is an outcrossing monocot species, it is often difficult to improve quantitative traits with conventional breeding and selection methods. Encouragingly, the production of transgenic switchgrass with unique genetic variations can be achieved by plant tissue culture approaches [17–20] and such transgenic switchgrass lines with traits of interest have shown promise for both agricultural and industrial purposes. For example, incorporation of a functional multigene pathway to produce polyhydroxybutyrate (PHB) in switchgrass has been demonstrated for biosynthesis of high-value biomaterials [21]. Other improvements of switchgrass by altering the lignin biosynthesis have been shown to increase saccharification efficiency and forage digestibility [22–25].

Relevant tissue culture techniques for switchgrass transformation were reported by Conger lab in the 1990s [26–31]. More recently, a protocol evaluating the tissue culture response from seed-derived calluses of 11 *Panicum* species was reported by Takamizo and co-workers [32]. At present, there are two types of grass embryogenic calluses that have been reported from callus induction. Type I calluses are white to yellowish in color, solid, slow growing, and are less regenerable. Type II calluses are dry in appearance, friable, fast growing, and are highly regenerable; however, the induction frequency of Type II calluses are generally lower than Type I calluses [28, 33–35]. In maize, the reported maintenance of regeneration ability of Type II callus is longer than Type I callus [36, 37]. In switchgrass, the Type II callus can be maintained for more than 6 months [38, 39]. In addition, long-term maintenance of caryopsis-derived Type I callus has recently been developed [40].

The successful transformation of the switchgrass cultivar Alamo was achieved in 2001 by particle bombardment using immature inflorescence-derived embryogenic callus [41]. Since 2002, an *Agrobacterium* (strain AGL1)-mediated genetic transformation of Alamo callus derived from mature caryopses has provided an alternative method for producing transgenic switchgrass under herbicide selection of Basta (bialaphos) [21, 42]. An *Agrobacterium*-mediated genetic transformation system using *Agrobacterium* strain EHA105 with hygromycin B selection has also been developed for the same switchgrass cultivar [20, 39, 43]. To increase the transformation efficiency, the *Agrobacterium*-mediated transformation technique was further improved to include infection under vacuum, co-cultivation in desiccation conditions, resting between co-cultivation and selection, and the supplementation with l-proline [39]. Later on, a high-efficiency switchgrass transformation was achieved by the selection of a pre-embryogenic “core” structure from the seed-derived callus [43]. However, due to the extremely low induction frequency of Type II callus, a new non-Murashige and Skoog (MS)-based medium, LP9, has been introduced from maize, which is useful to induce Type II callus from the inflorescences of the switchgrass Alamo 2 [38]. Another non-MS-based medium, NB_0_, was also developed as the basal medium to induce type II-like callus [44]. Recently, caryopsis-derived Type I embryogenic callus of the switchgrass cultivar Alamo has been developed for transgenic switchgrass production. The protocol for transformation of Type I embryogenic callus was further improved using 10 g l^−1^ glucose for 7 days at the co-cultivation stage and using 5 g l^−1^ casamino acid at the pre-culture step. This protocol is also applicable to the upland switchgrass cultivar Trailblazer [45, 46]. Although each protocol has its own advantages, a main challenge suggested in the literature is consistency and reproducibility [44].

Recently, our group has been working on projects expressing biocatalysts [47–49] and glycoside hydrolases (GHs) [50] in *Arabidopsis* and rice plants. In order to extend these activities to bioenergy plants, it is necessary to evaluate and build a simplified and reliable procedure to genetically modify the switchgrass plants for promoting biofuel production. The aims of this study are two-fold: first, we evaluated the major parameters of previously published methods for transgenic switchgrass production. These comparisons include processing time, tissue culture medium, *Agrobacterium* strain, and transformation approaches. Secondly, by taking the most effective steps from each of them, we consolidated and optimized the existing protocols into a new, effective protocol with improved ease and reproducibility.

## Results

### Evaluation of early tissue culture protocols

We first retrieved the available protocols from WorldCat (https://www.worldcat.org/) using a title search with the key words “switchgrass transformation”, returning 55 entries from the database. After reviewing the entries and manually removing redundant duplicates, we extracted nine original protocols for transgenic switchgrass production. To identify the differences between each protocol, the tissue culture process was generally broken down into five steps: (1) callus induction, (2) co-cultivation, (3) selection, (4) regeneration and (5) rooting. Detailed comparisons between these published protocols for producing transgenic switchgrass are listed in Additional file 1: Table S1. The comparative processing times for these protocols are listed in Table 1. In summary, the overall processing time for transgenic switchgrass production among the selected protocols ranges from 18 to 31 weeks and varies with the periods of callus induction (4–16 weeks), *Agrobacterium* co-cultivation (2–7 days), selection of transformants (4–16 weeks), shoot regeneration (4–6 weeks) and rooting (2–5 weeks) (Table 1). The optimization of a switchgrass tissue culture protocol in this study is based on evaluating and modifying each step of the published protocols (Table 1).

**Table 1:** Processing time among switchgrass transformation protocols reported in the literature and this study

	Callus induction I	Callus induction II	Co-cultivation	Selection	Regeneration	Light intensity (regeneration)	Rooting	Reference
1	N/A		3–7 days (27 °C)	8–16 weeks (27 °C)	4–6 weeks (27–28 °C)	80 μmol m^−2^ s^−1^	N/A	Somleva et al. [21, 42]
2	10 days (25 °C)	4 weeks	3 days	Until callus reaches 0.5 cm	2 weeks	N/A	2 weeks	Burris et al. [38]
3	8–12 weeks (24 °C)		2 days	5–8 weeks	4–6 weeks (25 °C)	140 μE m^−2^ s^−1^	4–5 weeks	Xi et al. [20]
4	6–8 weeks (26 °C)		2 days	6 weeks	3–4 weeks (25 °C)	140 μmol m^−2^ s^−1^	3–4 weeks (25 °C)	Li and Qu [39]
5	10 day-old seedling [White basal parts (5–8 mm)]		4 days (25 °C)	2–3 months	3–4 weeks (25 °C)	30 μmol m^−2^ s^−1^	N/A	Song et al. [51]
6	8–9 weeks (26 °C)		3 days (26 °C)	4–6 weeks (26 °C)	4–5 weeks (26 °C)	20001χ	2–3 weeks	Ramamoorthy and Kumar [44]
7	4 weeks (28 °C)		7 days (28 °C)	8 weeks (28 °C)	6 weeks (28 °C)	N/A	1–2 weeks (28 °C)	Ogawa et al. [46]
8	3–4 months (25 °C)		3 days (25 °C)	8 weeks	3 weeks (25 °C)	100 μmol m^−2^ s^−1^	3 weeks (25 °C)	Liu et al. [43]
9	4 weeks (28 °C)		7 days (22 °C)	8 weeks	6 weeks	N/A	N/A	Ogawa et al. [45]
10	4–6 weeks (26 °C)		3 days (26 °C)	7 weeks (26 °C)	3–4 weeks (26 °C)	100 μmol m^−2^ s^−1^	2–4 weeks (26 °C)	This study

*N/A* not applicable

### Seed sterilization and callus induction of parent line

The Alamo cultivar is the most popular and frequently used cultivar for establishing the transgenic protocols for switchgrass (Additional file 1: Figure S1A). For seed sterilization, we noticed sulfuric acid is often chosen as the scarifying agent [21, 28, 40, 42, 44–46]. To avoid the hazards of concentrated H_2_SO_4_, we chose commercially available Clorox^®^ bleach for seed scarification and sterilization. To remove the husks, the seeds were first immersed and gently stirred in a full-strength Clorox^®^ bleach solution (Additional file 1: Figure S1B) then rinsed five times with sterile distilled water (Additional file 1: Figure S1C). After seed sterilization, the dehusked seeds were ready for callus induction (Additional file 1: Figure S1D).

**Fig. 1:**
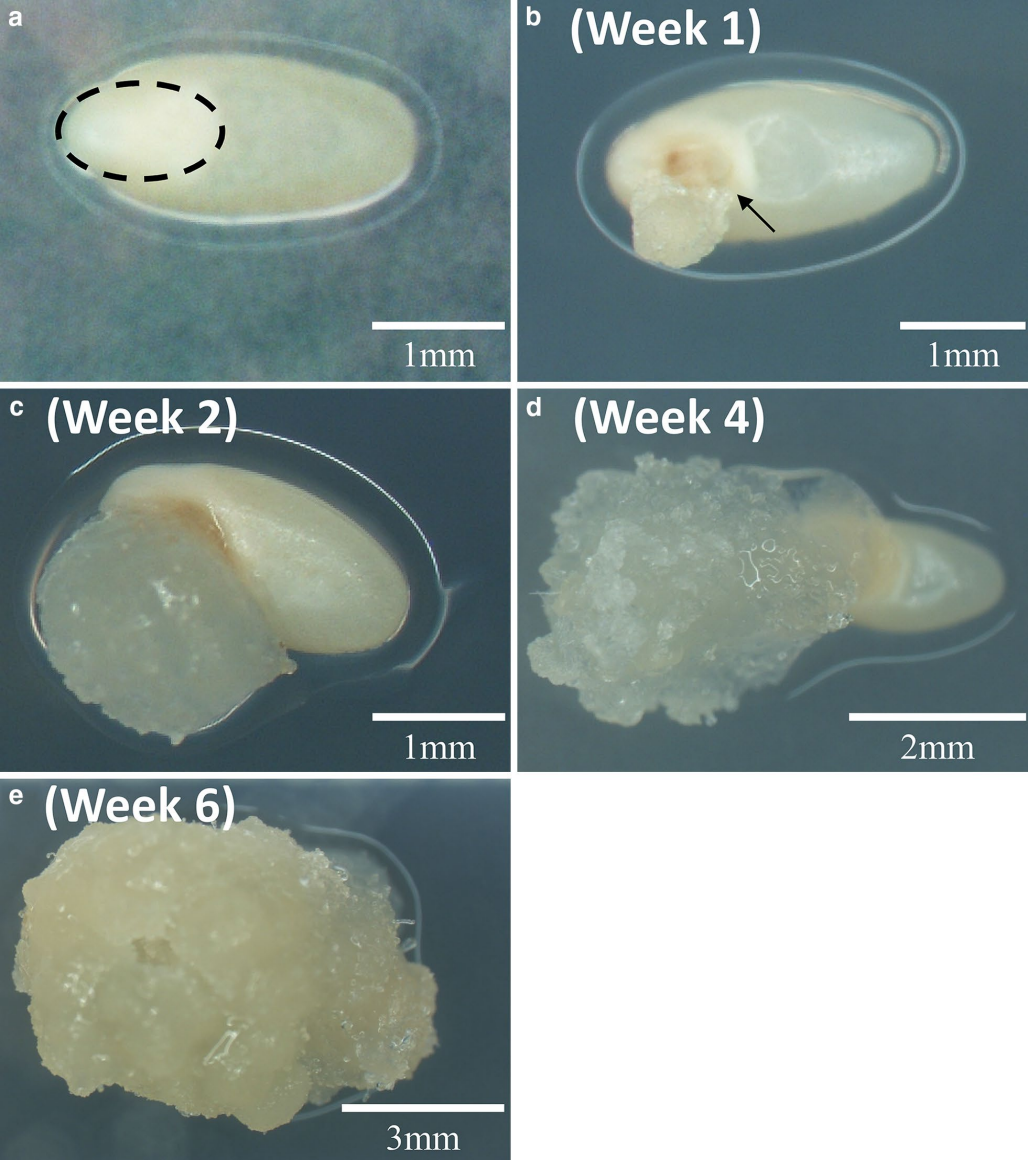
Callus induction of wild type switchgrass seeds. **a** Sterilized seeds on callus induction medium (CIM) at day zero. The embryo is indicated by the dashed oval. **b** Sterilized seeds on callus induction medium (CIM) at week one. The callus formation is initiated at the scutellum of the embryo (arrow), a tissue between the endosperm and coleoptile/coleorhiza. **c** Sterilized seeds on callus induction medium (CIM) at week two. **d** Sterilized seeds on CIM at week four. **e** Sterilized seeds on CIM at week six

For callus induction, the sterile seeds were placed on callus induction medium (CIM). The formation of callus can be observed rapidly in the first week on CIM at the scutellum (Fig. 1b). The volume of the rapid regenerative switchgrass calluses gradually increased during the following 3–5 weeks (Fig. 1c–e).

### Simplification of seed sterilization

Multiple sterilization steps, such as overnight incubation or additional disinfection, are often suggested [20, 21, 39, 42–46]. To simplify the seed sterilization step, we evaluated the two protocols which used bleach as their sterilization agent [39, 43]. However, because incompletely dehusked seeds were obtained after 2.5 h of seed sterilization by following the process in Liu et al. [43] (Additional file 1: Figure S2B and S2D), we decided to follow the seed sterilization step from Li and Qu [39] and worked to further improve the seed sterilization process from that starting point.

**Fig. 2:**
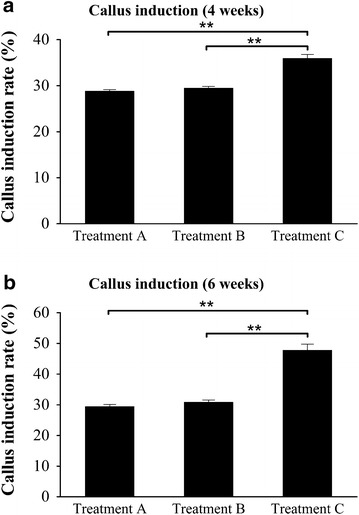
Callus induction rate of different sterilization treatments of the wild type seed. **a** Callus induction rate after 4 weeks on CIM. **b** Callus induction rate after 6 weeks induction. The callus induction rate of each treatment is represented as the mean of three replicates of 100 seeds per replicate. Error bars represent one standard error (SE) of the three replicates. Statistical testing was analyzed by one-way ANOVA followed by post hoc Bonferroni’s test (**p < 0.01) using Prism 5 (GraphPad Software Inc., La Jolla, CA)

The seed sterilization protocol was further examined using three seed sterilization treatments (i.e., A, B, C) with two sterilization approaches (one vs. two exposures to full-strength bleach) and two water incubation conditions (4 h vs. overnight) (see “Methods” section for details). The efficiency of callus induction for each treatment was compared. After 4 weeks of growth on CIM, the results showed that 10% more of the seeds induced callus formation under treatment C (Fig. 2a). Furthermore, the callus induction rate at 6 weeks for treatment C was about 20% higher (Fig. 2b). The results indicate that seeds in treatment C, with the milder bleach treatment and the shorter incubation time in water, were more effective at inducing callus (Fig. 2). Therefore, the seed sterilization was simplified to a single treatment with full-strength bleach for only 2.5 h followed by 4 h immersion in sterile water.

### Improvement of callus maintenance from parent line

Two different callus induction media are typically reported for the callus induction; Murashige and Skoog (MS)-based medium and N_6_ macronutrient/B_5_ micronutrient (NB)-based medium, Of note, NB-based medium has been successfully used in other monocot transformation protocol including rice [44]. NB-based medium was adopted as CIM in this study for monocot switchgrass because it has also been shown to promote the formation/production of highly friable calluses and to sustain the ability of calluses to regenerate [38, 44].

**Fig. 3:**
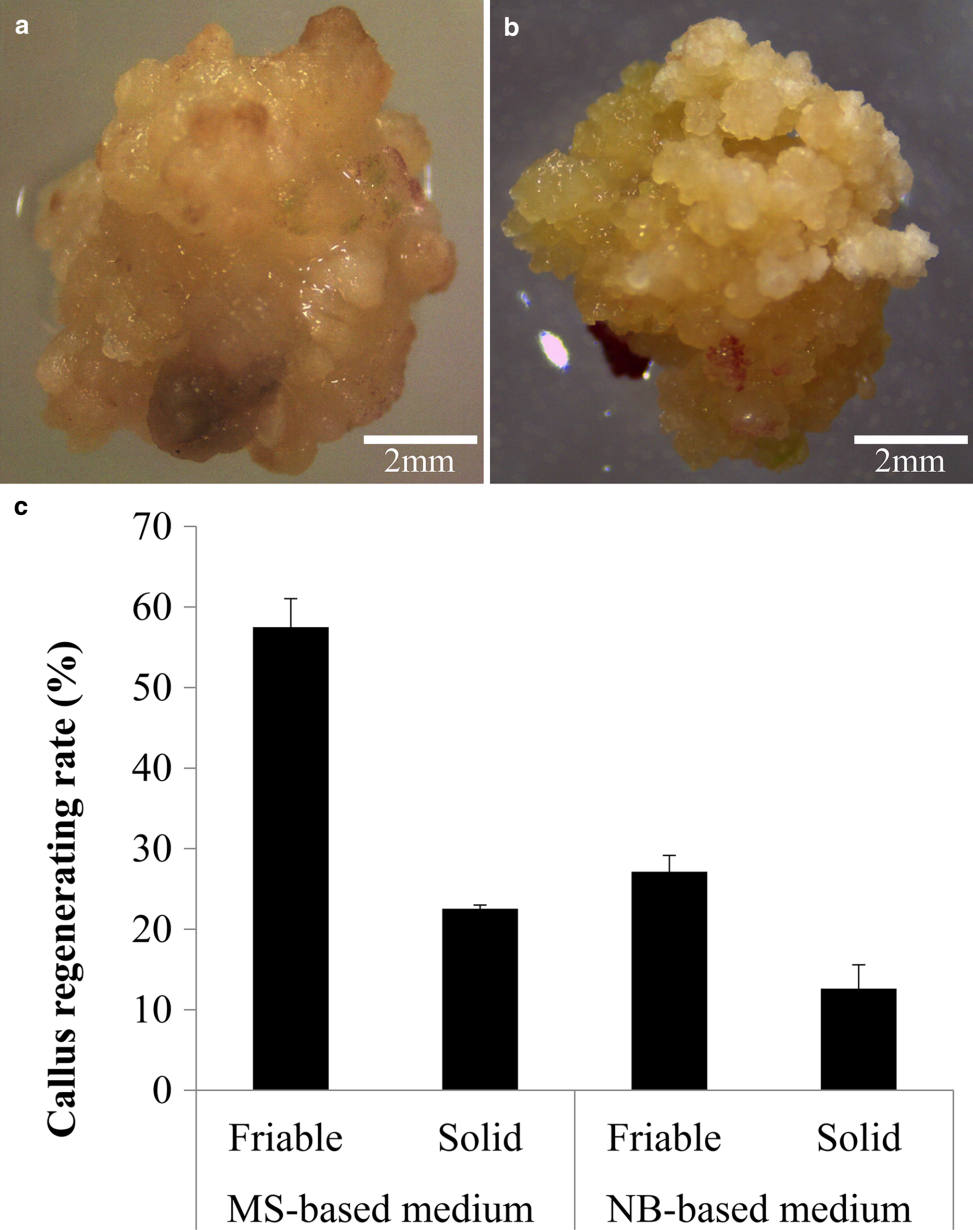
Plant regeneration of the two types of wild type switchgrass callus and the regeneration rate on MS-based and NB-based media. **a** The overall look of solid (Type I) switchgrass callus. **b** The overall look of friable (Type II) switchgrass callus. **c** Regeneration rate of Type I and Type II calluses on MS-based and NB-based media. The values of each treatment represent the means of the three replicates of 20–30 pieces of callus per replicate. Error bars represent one SE of the three replicates

Using the NB-based CIM medium, both types of calluses could be induced from mature switchgrass seeds, Type I callus (Fig. 3a) and Type II callus (Fig. 3b). Approximately 30% of the induced calluses were Type II, which is similar to the rate of Type II callus induction reported in previous studies using NB-based medium. To further increase and improve the induction of type II callus, we adopted a recent procedure that led to the observation of a shell-core structure in switchgrass induced calluses reported in Liu et al. [43]. Liu et al. [43] also reported that the majority of calluses with separated cores gradually developed into type II callus. Therefore, to promote the development of type II calluses in this study, the protocol was optimized by manually dissecting the induced calluses to about 0.2 cm pieces when transferred to fresh CIM to release the core from the induced calluses (see “Methods” section for details). Remarkably, by actively dividing and propagating the friable calluses, 100% Type II callus induction can be achieved after 8–10 weeks (Additional file 1: Figure S3). Moreover, Li and Qu [39] described that the regenerated plants were albino after calluses were in culture for 14 months. In this study, since the calluses were not maintained for more than 6 months, we did not observe chlorotic or sterility among the regenerated plants.

### Optimization of plantlet regeneration from callus of parent line

Three kinds of plant hormones, gibberellic acid (GA_3_), kinetin and 6-benzylaminopurine (BAP), are frequently used for switchgrass calluses regeneration. Alternatively, the addition of α-naphthaleneacetic acid (NAA) and indole-3-acetic acid (IAA) have also been recently reported [39, 44]. To simplify the current protocol, we first added GA_3_, NAA and BAP in the same NB-based medium, which is a simpler approach using a single basal medium described by Ramamoorthy and Kumar [44]; however, there is a relatively high incidence of regeneration failure for switchgrass calluses when NB-based medium is supplemented with these hormones (Additional file 1: Figure S4). Therefore, we attempted to optimize the plant regeneration medium for switchgrass calluses, as described below.

We chose a different set of hormone supplements, which is reported in Ramamoorthy and Kumar [44] for switchgrass plant regeneration. Both MS- and NB-based media were chosen to evaluate their abilities to regenerate switchgrass calluses and the plant hormones (IAA, NAA, BAP and kinetin) were added as REG medium supplements. First, the two types of switchgrass calluses were selected based on the distinct features of type I and type II calluses (Fig. 3a, b). The plant regeneration results show that friable calluses (Type II) have better regenerative ability than solid calluses (Type I) (Fig. 3c), Moreover, compared to the NB-based medium, MS-Based medium showed a two-fold increase in regeneration rate for both types of calluses (Fig. 3c).

### Consolidation and optimization of hygromycin B dosage for selection

For transgenic switchgrass production, hygromycin phosphotransferase gene (*hph*) was used as an effective selection marker in 6 of the 9 published protocols (Additional file 1: Table S1). Several desirable hygromycin B concentrations for the selection of transformed calluses have been previously examined and reported as 50 mg l^−1^ for 2–3 months [51], 60 mg l^−1^ for 2 months [38], 75 mg l^−1^ for ~ 4–8 weeks [20, 44], 50–100 mg l^−1^ for 6 weeks [43] and 100–200 mg l^−1^ for ~ 6 weeks [39]. As known that the commercial source of hygromycin B can affect its potency [52], after evaluating the antibiotic strength and the selection period among these protocols, we optimized the hygromycin B selection using 50–100 mg l^−1^ for a 6-week-peroid (Table 2; See “Methods” section for details), which represents a moderate dosage of hygromycin B selection combined with a rapid selection approach for transformed calluses modified from Liu et al. [43].

**Table 2:** Tissue culture medium composition used in this protocol

Purpose	Medium name	Composition
Callus induction	CIM	NB_0_ medium (pH 5.8) (3 mg l^−1^ 2,4-d + 0.2 mg l^−1^ BAP + 500 mg l^−1^ l-proline)
Callus resting	CRM	MS medium (pH 5.8) (5 mg l^−1^ 2,4-d + 1 mg l^−1^ BAP + 2 g l^−1^ l-proline + 200 mg l^−1^ Timentin)
Callus selection	CSM	MS medium (pH 5.8) (5 mg l^−1^ 2,4-d + 1 mg l^−1^ BAP + 2 g l^−1^ l-proline + 200 mg l^−1^ Timentin) (50–100 mg l^−1^ hygromycin B)
Regeneration	REG	MS medium (pH 5.8) (2 mg l^−1^ BAP + 1 mg l^−1^ IAA + 1 mg l^−1^ kinetin + 1 mg l^−1^ NAA + 500 mg l^−1^ l-proline + 200 mg l^−1^ Timentin) (20 mg l^−1^ hygromycin B)
Rooting	RM	1/2 MS salts + 30 g l^−1^ maltose (pH 5.8) (200 mg l^−1^ Timentin + 50 mg l^−1^ hygromycin B)

NB_0_ medium, NB Basal Medium + 30 g l^−1^ maltose + 300 mg l^−1^ casein enzymatic hydrolysate (CH) + 6.5 g l^−1^ agar MS medium, MS Basal medium with vitamins + 30 g l^−1^ maltose + 6.5 g l^−1^ agar or 3 g l^−1^ phytagel (in REG)

To reduce the escape of non-transformed WT seedlings, we examined the dose-dependent inhibition of rooting (killing curve) using WT switchgrass seedlings. A range of hygromycin B concentrations (0, 20, 40 and 80 mg l^−1^) were added in the rooting medium (RM) (Table 2) to test the effect of rooting inhibition. The results show that hygromycin B can inhibit the normal growth of switchgrass at concentrations above 40 mg l^−1^, while rooting of WT seedlings was totally inhibited when hygromycin B was supplemented at 80 mg l^−1^ (Additional file 1: Figure S5). Therefore, we choose 50 mg l^−1^ hygromycin B as optimal for the rooting of transgenic switchgrass, which is also consistent with the effective working concentration reported in some protocols [39, 43, 44].

### *Agrobacterium* preparation

Three plasmids were acquired or constructed for this protocol to evaluate *Agrobacterium*-mediated transformation of switchgrass. These were pCAMBIA1305.2, pCAMBIA-EV (empty vector) and pCAMBIA-RFP (red fluorescence protein). The plasmid pCAMBIA1305.2 was used to overexpress a hygromycin phosphotransferase gene (*hph*) and a *GUSPlus* gene fused with the glycine-rich protein (GRP) signal peptide for secretion with each gene driven by a CaMV35S promoter in opposite direction (www.cambia.org). The pCAMBIA-EV plasmid was used to produce the hygromycin B-resistant transgenic switchgrass plants, which serve as controls lacking the *GUSPlus* expression cassette of pCAMBIA1305.2. The pCAMBIA-RFP was generated by inserting pporRFP expression cassette from pANIC6A [53] into pCAMBIA-EV, which is useful to validate the switchgrass transformants by direct visualization of red fluorescence. The three plasmids (pCAMBIA-EV, pCAMBIA1305.2 and pCAMBIA-RFP) were introduced into *Agrobacterium* (EHA105) and the *Agrobacterium* transformants were confirmed by PCR using specific primer sets (Additional file 1: Table S2) to detect each gene (*hph*, *RFP* and *GUSPlus*) in the corresponding transformants before conducting *Agrobacterium*-mediated genetic transformation (Additional file 1: Figure S6).

### Simplification of *Agrobacterium*-mediated transformation and callus selection

After evaluating the previous protocols for *Agrobacterium*-mediated transformation, we chose to follow the Li and Qu [39] protocol because they report a relatively high efficiency for producing transgenic switchgrass (50%) (Additional file 1: Table S1). However, as noted by Li and Qu [39], choosing the right callus type(s) is crucial for high frequency transformation. Both types of calluses have been reported to successfully produce transgenic switchgrass [39, 45] and we have optimized the MS-based REG medium for enhanced regeneration of both types of calluses (Fig. 3c). To further simplify the transformation process, we performed the *Agrobacterium*-mediated transformation on the induced calluses without pre-selection of the right callus type(s).

For transgenic switchgrass production, calluses with 0.3–1 cm in diameter were selected. All calluses were pre-cultured on fresh CIM for 2 days followed by immersion in a prepared *Agrobacterium* solution harboring the specific plasmid (see “Methods” section for details and Additional file 1: Figure S7A). The calluses immersed in *Agrobacterium* suspension were subjected to vacuum infiltration (Additional file 1: Figure S7B), agitation, 2 days of desiccation (Additional file 1: Figures S7C and S7D), followed by resting on callus resting medium (CRM, Table 2) for 7 days before transferring the callus to callus selection medium (CSM).

**Fig. 4:**
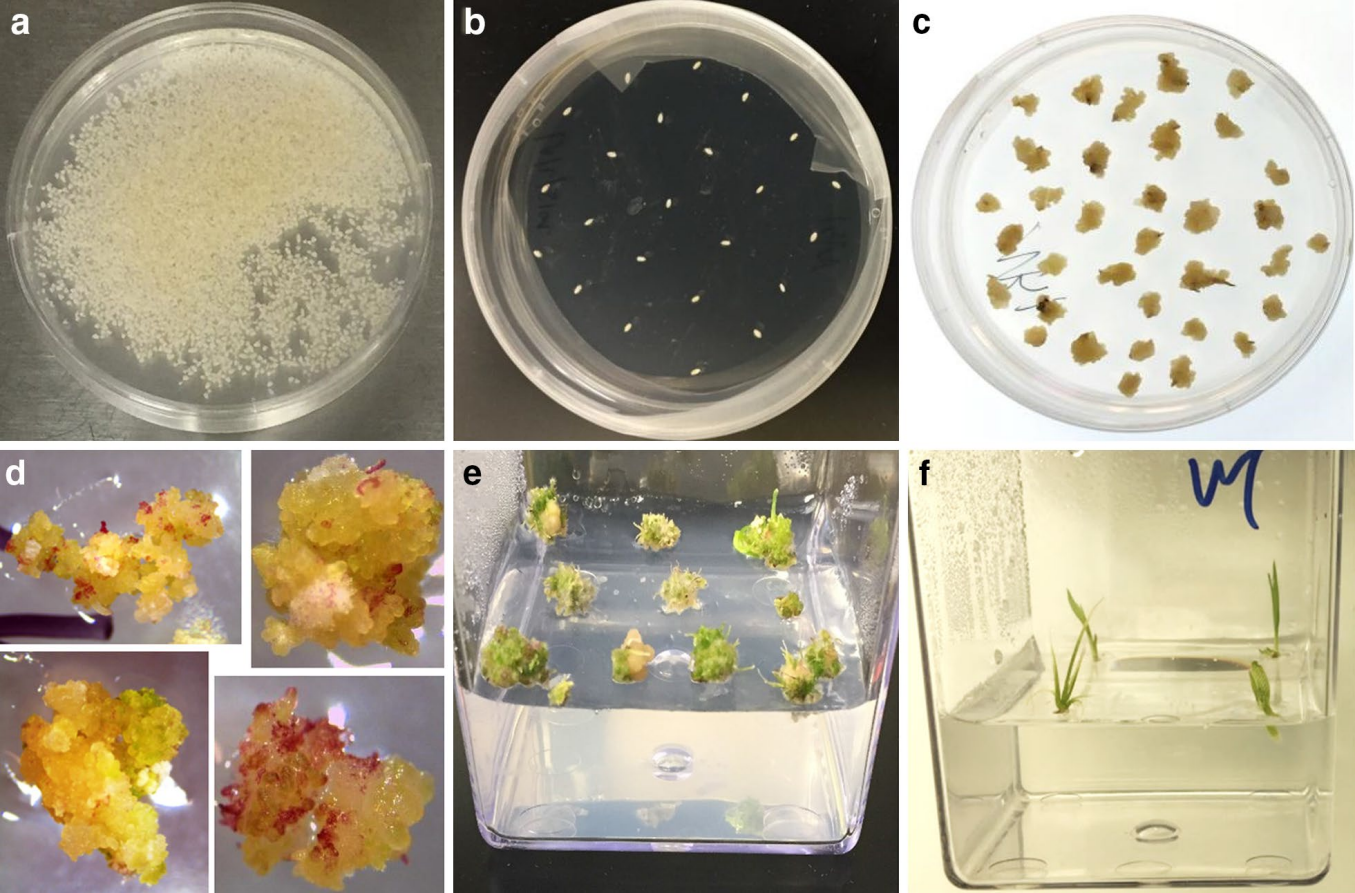
Overall tissue culture protocol for wild type switchgrass regeneration. **a** The sterilized seeds were transferred from flasks to Petri dishes. **b** Sterilized seeds were arranged on CIM for callus induction with 20–25 seeds per dish. **c** Callus formation after 6 weeks of callus induction. **d** Plant regeneration on REG under light intensity of 100 µmol m^−2^ s^−1^. **e** Regenerated shoots were observed after 2–4 weeks on REG. **f** Shoots (1–1.5 cm) were excised and transferred onto RM

The selection of putative transgenic calluses was modified from Liu et al. [43]. The selection strategy is composed of two rounds of 50 mg l^−1^ hygromycin B and one round of 100 mg l^−1^ hygromycin B over a 6-week period, a shorter and lower antibiotic usage selection approach than previous protocols (Additional file 1: Table S1). After selection, the hygromycin B-resistant calluses were transferred to REG medium supplemented with 20 mg l^−1^ hygromycin B (Table 2) for plant regeneration. The regenerating calluses can be distinguished by the red–purple pigmentation (anthocyanin induction), green nodular appearance, and emergence of apparent shoot meristems (Fig. 4d). Once the shoots elongated to ~ 1.0–1.5 cm, the regenerated transgenic shoots were transferred to RM for rooting (Fig. 4f).

### Molecular analysis of transgenic plants

Genomic DNA was extracted from leaf tissues of putative transgenics and PCR was conducted to detect the presence of the transgenes using specific primer sets of the corresponding transgenic lines (Additional file 1: Table S2). Extracting genomic DNA to confirm the transgenes had been reported using 2–4-week-old seedlings [46], greenhouse-grown T_0_ plants [20, 51], or T_1_ plants [39], Although we can detect the presence of the *hph* gene in 3-month-old greenhouse-grown pCAMBIA-EV transgenic lines by genomic DNA PCR (Additional file 1: Figure S8), for a more rapid primary screening of the putative transformants, we subjected young leaves from 2-week-old seedlings to a rapid primary screen to identify putative switchgrass transformants. To avoid false positive PCR results, we used a higher concentration of Timentin (200 mg l^−1^) to suppress the overgrowth and contamination of *Agrobacterium* [43] and we only considered clear and strong PCR bands as true transgenics [20, 46].

**Fig. 5:**
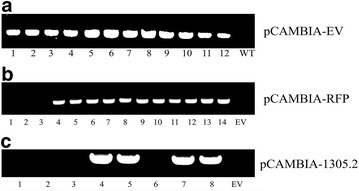
The primary screening for the presence of transgene in individual putative transgenic switchgrass plants using genomic DNA PCR. **a** Amplification of *hph* gene in pCAMBIA-EV transformed transgenic switchgrass. **b** Amplification of *pporRFP* gene in pCAMBIA-RFP transformed transgenic switchgrass. **c** Amplification of *GUSPlus* gene in pCAMBIA-1305.2 transformed transgenic switchgrass

**Fig. 6:**
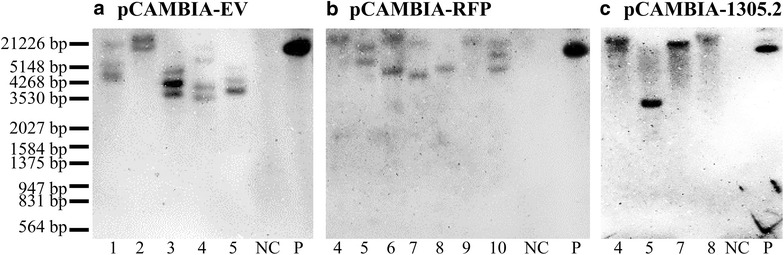
Transgene integration in transgenic switchgrass lines using Southern blot analysis by *hph* probe. **a** pCAMBIA-EV transformed transgenic switchgrass. **b** pCAMBIA-RFP transformed transgenic switchgrass. **c** pCAMBIA-1305.2 transformed transgenic switchgrass. *NC* negative control, *P* positive control using *Hind*III-linearized pCAMBIA-1305.2

The genomic DNA PCR results are shown in Fig. 5 and the overall transformation outcomes for each construct are listed in Table 3. In summary, about 70% of the calluses survived 6 weeks of hygromycin B selection, ~ 21–27% of calluses regenerated on MS-based REG medium, and 80–92% of regenerated calluses produced roots and grew into transgenic plants (Table 3). For pCAMBIA-EV, 12 putative transgenics were produced and all of these transgenics tested positive for the *hph* gene, indicating a selection efficiency of 100% (Fig. 5a). For pCAMBIA-RFP, 11 of the 14 putative RFP transgenics tested positive (78.5%) (Fig. 5b), while pCAMBIA-1305.2 transgenics was 50% (4 of 8 transgenics were confirmed positive for the presence of the *GUSPlus* gene) (Fig. 5c). Moreover, integration of the transgenes was confirmed by Southern blot analysis using 5 μg of overnight digested genomic DNA (Additional file 1: Figure S9). One to four copies of each transgene were estimated to be integrated into each transgenic line as a result of *Agrobacterium*-mediated transformation (Fig. 6).

**Table 3:** *Agrobacterium*-mediated transformation of switchgrass Alamo calluses without pre-selection of calluses types

Vector	Experiment	No. of calluses inoculated	No. of hygromycin resistant callus after 6 weeks selection	No. of regenerated callus	No. of rooting seedling	No. of plants positive for genomic DNA PCR	Selection frequency (%)
pCAMBIA-EV	1	26	19	4	4	4	
	2	31	22	4	4	4	
	3	33	19	5	4	4	
	Total	90	60	13	12	12	100.0
pCAMBIA-RFP	1	26	18	4	5	2	
	2	34	23	6	5	5	
	3	35	22	7	4	4	
	Total	95	63	17	14	11	78.6
pCAMBIA1305.2	1	20	19	5	4	1	
	2	30	23	5	4	3	
	Total	50	42	10	8	4	50.0

Selection efficiency: number of genomic DNA PCR positive switchgrass transformants divided by the regenerated and rooted hygromycin B resistant switchgrass seedlings

To further verify the expression of the transgene, we extracted the RNA from 3-month-old greenhouse-grown transgenic switchgrass plants (Lane 1 in Additional file 1: Figure S10) to perform reverse transcription PCR (RT-PCR). The RT-PCR results showed the expression of the transgene in the corresponding switchgrass transgenic plants (Lane 2 in Additional file 1: Figure S10), supporting the results of the initial genomic DNA PCR screen (Fig. 5). Furthermore, to eliminate the possible false-positive result from genomic DNA contamination in RT-PCR (Lane 2 in Additional file 1: Figure S10), we performed the same PCR reactions on the isolated RNA. There are no PCR products using RNA as template, which indicates the successful transgene expressions and no contamination of the genomic DNA from the *Agrobacterium* or transgenic plants in the extracted RNA (Lane 3 in Additional file 1: Figure S10).

### Functional analysis of the transgenic switchgrass

**Fig. 7:**
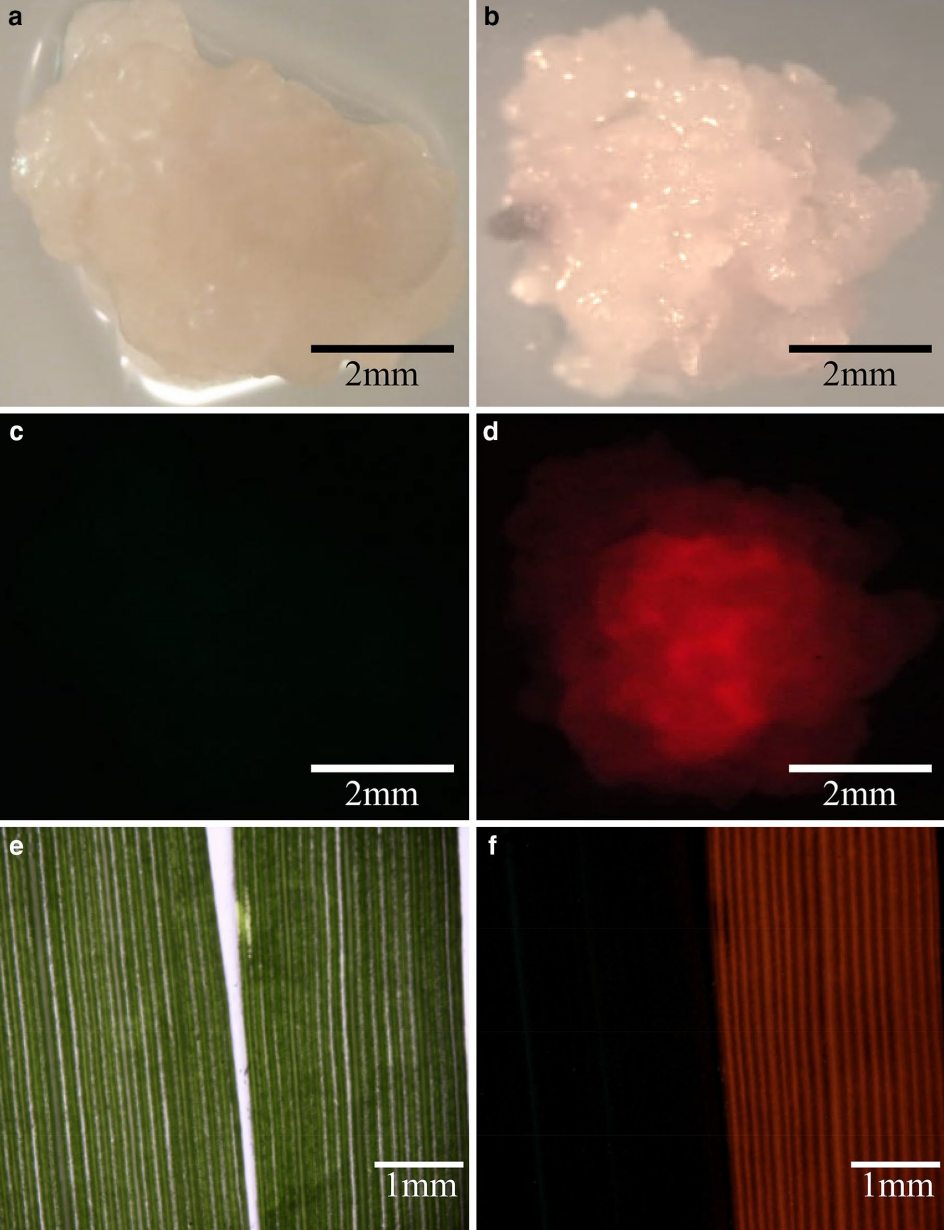
Detection of pporRFP expression in pCAMBIA-EV and pCAMBIA-RFP transformed switchgrass. **a** Bright field image of pCAMBIA-EV callus. **b** Bright field image of pCAMBIA-RFP transformed callus. **c** Epi-fluorescence image of pCAMBIA-EV callus. **d** Epi-fluorescence image of pCAMBIA-RFP transformed callus. **e** Bright field image of switchgrass leaves (Left: pCAMBIA-EV transgenic line; right: pCAMBIA-RFP transgenic line). **f** Epi-fluorescence image of switchgrass leaves (Left: pCAMBIA-EV transgenic line; right: pCAMBIA-RFP transgenic line)

Green fluorescent protein (GFP) and red fluorescent protein (RFP) reporters have been successfully used in switchgrass to monitor the transformation process [39, 44, 53]. In this protocol, we adopted *pporRFP* to further validate the results of primary screening analysis (Fig. 5b). The *pporRFP* expression cassette is driven by the switchgrass ubiquitin 1 promoter (PvUbi1), a constitutively active promoter in all switchgrass tissues (leaf, flower, stem, root and callus) [54]. Using a fluorescent stereo-dissection microscope, the expression of the pporRFP can be observed from the red fluorescence at the callus stage from 2 to 6 weeks after the transformation (Fig. 7d), when compared to the pCAMBIA-EV control (Fig. 7c). After plant regeneration, the red fluorescence was detected in the leaves of all genomic PCR-confirmed pCAMBIA-RFP switchgrass transformants (Fig. 7f); and as well as in the roots (Additional file 1: Figure S11). Different expression levels of pporRFP protein can also be observed by the intensity of pporRFP fluorescence in the protein extract of pCAMBIA-RFP switchgrass transgenics (Additional file 1: Figure S12).

**Fig. 8:**
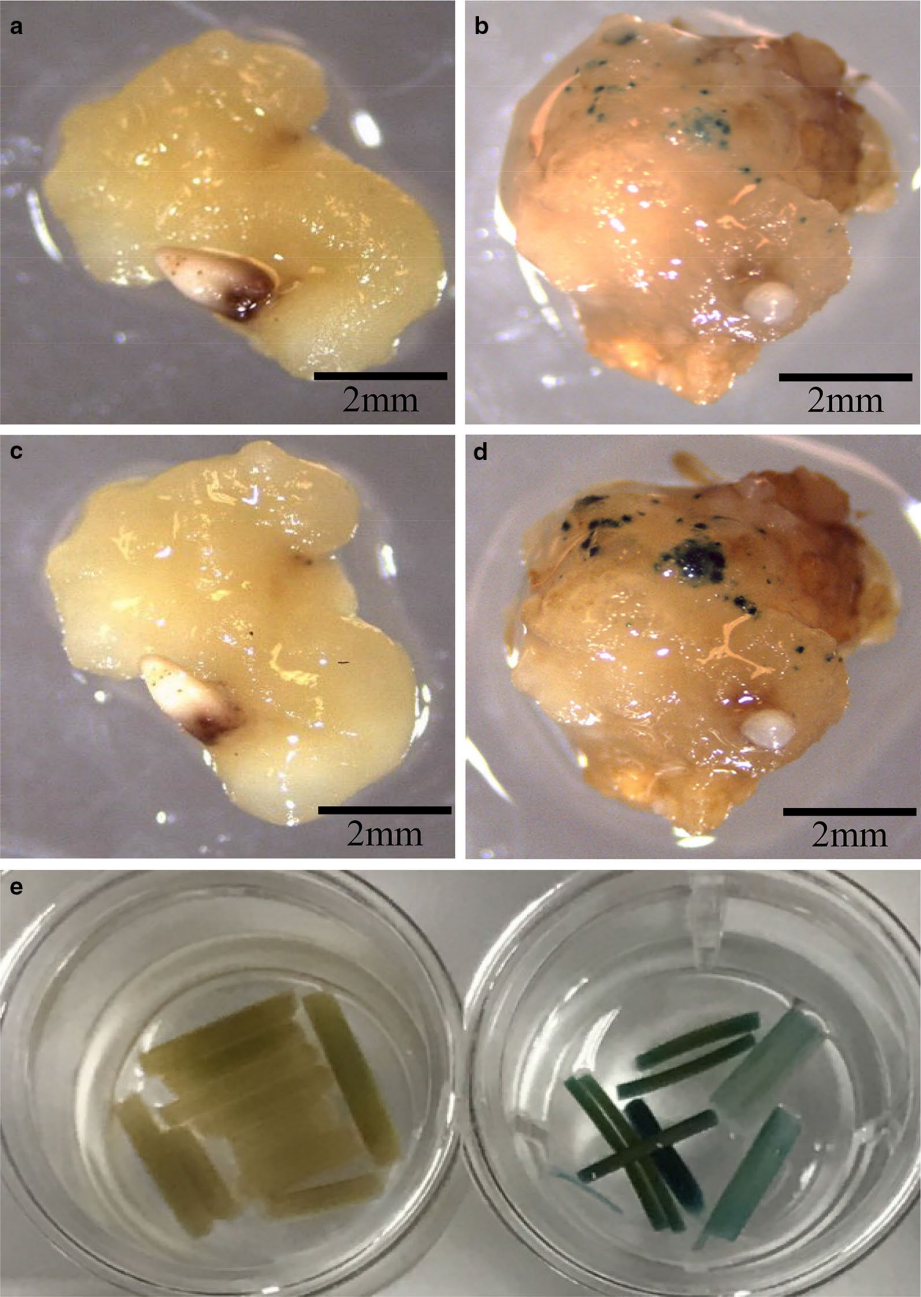
Histochemical GUS staining of pCAMBIA-EV and pCAMBIA1305.2 transformed switchgrass. **a** pCAMBIA-EV callus incubated in GUS staining solution at 37 °C for 4 h. **b** pCAMBIA1305.2-transformed callus incubated in staining solution at 37 °C for 4 h. **c** pCAMBIA-EV callus incubated in staining solution at 37 °C overnight. **d** pCAMBIA1305.2-transformed callus incubated in staining solution at 37 °C overnight. **e** Switchgrass stems and leaves incubated in staining solution at 37 °C overnight (Left: pCAMBIA-EV transgenic line; right: pCAMBIA-1305.2 transgenic line)

The β-glucuronidase (GUS) reporter system [55] was also included to validate the primary screening results of the putative transformants (Fig. 5c). Histochemical GUS staining of switchgrass tissue at different stages was useful to confirm the functionality of *GUSPlus* and to guide the transformation process for higher selection efficiency. At the callus stage, GUS staining can be detected after 4 h of incubation in the staining solution at 37 °C (Fig. 8b) with signal intensifying after overnight incubation (Fig. 8d). In the control callus, no GUS signal was detected from either the 4 h or overnight incubations (Fig. 8a, c). Moreover, the 35S promoter-driven *GUSPlus* overexpression can be detected by histochemical GUS staining in leaves and stem tissues of the pCAMBIA-1305.2 transgenic switchgrass plants (Fig. 8e).

## Discussion

### Consolidation of seed sterilization procedure

Several of the published seed sterilization protocols used 60% sulfuric acid (H_2_SO_4_) for seed dehusking [21, 28, 40, 42, 44–46] and 50–100% bleach for surface sterilization [20, 38, 39, 43]. It has also been reported that dehusked seeds are more effectively surface sterilized than non-dehusked caryopses, given that dehusking enables 80–90% of the seeds to germinate without contamination [51]. Moreover, some protocols have suggested an additional overnight incubation in sterile water following the sterilization for seed imbibition [39, 43, 46]. However, we had observed a higher incidence of damaged embryos and lower callus induction rate after multiple steps of seed sterilization or overnight incubation. Therefore, the seed sterilization process was optimized to a single step that applied full strength bleach for only 2.5 h without overnight incubation. This approach avoids the potential hazards of sulfuric acid and is a simpler 1 day sterilization process.

### Consolidation of callus induction and maintenance methods

Callus induction medium (CIM) was modified according to previous protocols [39, 43, 44]. Among the published protocols (Table 1), six used MS-based medium [20, 39, 42, 45, 46, 51], and two utilized NB-based medium [38, 44]. The type II callus induction rates on MS-based medium were reported to be 10–15% [39, 43]; while NB-based medium had superior Type II callus induction rates of about 33% [38, 44]. NB-based medium also demonstrated an increased ability to maintain plant regeneration [38]. Based on these facts, we selected NB-based medium as the optimal medium for callus induction (Table 2).

Our results showed clear evidence that vigorously dividing switchgrass calluses originate from the scutellum of mature switchgrass seeds (Fig. 1b), which was generally believed to be competent for *Agrobacterium*-mediated transformation in rice [56]. We improved seed sterilization to increase callus induction rate up to 20% over previous protocols (Fig. 2). However, the sterilization step can still be further optimized to balance the rate of seed survival versus contamination. Furthermore, in this protocol, the induced calluses were intentionally maintained by releasing the core of the callus [43]. In addition, use of the recently developed switchgrass line (HR8) with its high seed germination (82%) and SE callus production capacities (84.9%) may further increase the Type II callus induction rate [57].

### Consolidation of plant regeneration protocols

Plant regeneration using MS-based medium versus NB-based medium was also investigated in this study. The regeneration of callus was successfully demonstrated using MS-based medium supplemented with GA_3_ [21, 42, 45, 46, 51], kinetin [20], BAP [38], or NAA, GA_3_ and BAP [39, 43]. Besides the MS-based medium, there was only one report that had shown using the NB-based medium for plant regeneration. In this study, MS-based medium and NB-based medium were used to evaluate their regeneration abilities for both types of calluses (Fig. 3a, b) since they had been reported to produce transgenic switchgrass plants successfully. Our results reported here show that Type II calluses have higher regenerative ability than Type I calluses, which is consistent with the previous reports (Table 1). Compared to the NB-based medium, MS-based medium has better regeneration rates for the both types of callus (Fig. 3c). Therefore, we optimized the REG medium to use MS-based medium supplemented with plant hormones (IAA, NAA, BAP and kinetin).

### Factors contributing to transformation efficiency

#### Choice of switchgrass cultivars

Biolistic particle bombardment has been successfully employed to produce transgenic switchgrass [41]. However, more recent protocols have adopted *Agrobacterium*-meditated transformation [20, 38–40, 42, 44–46, 51]. In general, the transformation efficiency (TE) for the lowland Alamo switchgrass using *Agrobacterium*-meditated transformation can reach 56.6–72.8% [39, 43, 45]. Several attempts have been made to generate transgenic switchgrass using upland switchgrass cultivars. However, no regenerated plants were obtained using upland octoploid cultivar CIR [51], only 8% TE were reported for upland tetraploid cultivar Dacotah [43] and 7.5% successful transformation rates for upland octoploid cultivar Trailblazer [45]. The upland switchgrass is generally more recalcitrant to *Agrobacterium*-meditated transformation. They display lower plant regeneration rates, a tighter, stronger shell structure of the callus, and loss of regeneration ability during the transformation process [43, 45, 51].

#### Choice of *Agrobacterium* strains

There are several factors that can influence *Agrobacterium*-mediated TE for transgenic switchgrass production including *Agrobacterium* strain, the presence of acetosyringone during the inoculation, and the co-cultivation approach. The five strains of *Agrobacterium* reviewed for their effect on TE in this study were AGL1, GV3101, LBA4404, EHA101, and EHA105 (Additional file 1: Table S1). EHA105 and AGL1 have higher transformation efficiencies among the strains used in published protocols [20, 51, 58] and EHA105 was chosen as the most common strain used for transgenic switchgrass production (Additional file 1: Table S1).

*Agrobacterium*-mediated transformation usually results in a lower copy number of transgenes integration [59]. Protocols typically report a range of 1–5 copies of transgenes inserted into the switchgrass genome (Table 1). Most published protocols used 15–20 μg of overnight-digested genomic DNA to perform Southern blot analysis (Table 1); however, to lower the required amounts of genomic DNA, two concentrations of genomic DNA were examined, which are 5 and 10 μg. The result showed 5 μg of genomic DNA is sufficient for Southern blot analysis using chemiluminescence detection method (Additional file 1: Figure S9). By following the procedure described in Ramamoorthy and Kumar [44], we estimated that 1–4 transgene copies were inserted into switchgrass genome (Fig. 6).

#### Choice of *Agrobacterium*-mediated transformation

The most variable part of the published protocols was the approach for co-cultivation of *Agrobacterium* strains with the plant tissues. Additional steps have been developed to enhance transformation efficiency, such as placing calluses on CIM for 3–7 days [38, 42, 58], placing them on co-cultivation medium for 3–7 days [44, 46, 51, 57], coupling with vacuum infiltration (0.53–0.79 atm) [20, 38, 39, 43], desiccation on filter paper [20, 39, 43], or providing an additional resting step before callus selection [39, 43]. By following the co-cultivated approach described by Li and Qu [39], we can routinely produce transgenic switchgrass plants with a selection efficiency of 50–100% (Table 3). Desiccation treatment has been often used in plant tissue culture to improve regeneration [60, 61], indicating it is a beneficial treatment for producing transgenic monocot plants.

Due to the challenges of transforming the upland switchgrass cultivars and the limited histological understanding of upland switchgrass calluses, we chose to focus on the more widely accepted model lowland switchgrass cultivar Alamo for this protocol optimization. However, the differences among each co-cultivation approach should be further investigated, which may help to develop a better co-cultivation approach for upland switchgrass cultivars.

## Conclusions

We report an improved transgenic switchgrass protocol with a step-by-step visualization of the process using the lowland Alamo switchgrass cultivar. This protocol was developed based on detailed evaluation and experimentation with previously published protocols (Table 1 and Additional file 1: Table S1). In this study, we provide a streamlined work-flow (Figs. 1 and 4 and Additional file 1: Figures S1 and S7) and a list of consolidated tissue culture media (Table 2). In addition, we simplified the seed sterilization procedure to a single step for scutellum-derived callus induction (Fig. 2) and developed a modified MS-based REG medium for the regeneration of both types of switchgrass calluses. In addition, we are able to enhance the regeneration rate of both types of switchgrass callus using the modified MS-based REG medium, which can reduce the prerequisite for Type II callus for *Agrobacterium*-meditated transformation.

We demonstrate the successful and improved management of the switchgrass tissue culture using our optimized protocol, which requires 2–3 months from seed-derived callus to plantlet regeneration. Transgenic switchgrass production is achievable within 4–6 months including an additional 7 weeks of *Agrobacterium*-meditated transformation and selection, which is a relatively time and cost effective protocol for producing transgenic switchgrass compared to published protocols (Table 1). Our optimizations of current protocol include single step seed sterilization (1 day), improved type II callus induction by releasing the cores of calluses by actively dividing the induced calluses, improved plant regeneration using modified MS-based regeneration medium, and a desirable rooting medium for transgenic switchgrass selection. Using this protocol, the switchgrass transformants were confirmed by genomic DNA PCR to have selection efficiency of 50% up to 100% (Fig. 5 and Table 3), and the expression of the transgenes is readily detected by RT-PCR in the switchgrass transgenic plants (Additional file 1: Figure S10).

This study has selected and fine-tuned the previously published protocols to develop a simplified and improved process for transgenic switchgrass generation that can facilitate the tailoring of switchgrass biomass for a specific trait-of-interest. Recently, the CRISPR/Cas system has been successfully demonstrated in switchgrass for genome editing [62]. The protocol improved and validated in this study should benefit the efficient application of current genome editing biotechnologies in switchgrass [63].

## Methods

### Chemicals

NB basal medium (N492), MS basal medium with vitamins (M519), maltose (M588), 2, 4-dichlorophenoxyacetic acid (2, 4-d, D295), indole-3-acetic acid (IAA, I885), α-naphthaleneacetic acid (NAA, N600), 6-benzylaminopurine (BAP, B800), kinetin (K750), l-proline (P698) were purchased from PhytoTechnology Laboratories (Lenexa, KS). Hygromycin B from *Streptomyces hygroscopicus* (H-270), timentin™ (T-104) and X-Gluc (G1281C) were purchased from Gold Biotechnology (St. Louis, MO). All other chemicals were purchased from Sigma-Aldrich (St. Louis, MO).

### Plant material, tissue culture and conditions

Wild type (WT) switchgrass (*P. virgatum*) cultivar Alamo seeds were purchased from Ernst Conservation Seeds, Inc. (Meadville, PA) and stored in the dark at 25 °C and ambient humidity. All tissue culture procedures were conducted under sterile conditions and micro-dissecting instruments were sterilized by hot bead dry sterilizer, Germinator 500 (CellPoint Scientific, Gaithersburg, MD). Callus induction, growth and hygromycin B selection were incubated at 26 °C in the dark. Regeneration of transformed calluses and growth of switchgrass transformants were carried out in a growth chamber (E-41HO, Percival Scientific, Perry, IA) at 26 °C, under a 16 h light/8 h dark cycle with a light intensity of 100 µmol m^−2^ s^−1^. Two weeks after rooting, the switchgrass transgenics were transferred to pots filled with Metro-Mix 360 (SunGro Horticulture, Agawam, MA).

### Seed sterilization and callus induction from parent line

Three treatments (A, B, C) were modified from published protocols (Additional file 1: Table S1). For treatment A, ~ 6 g of seeds were surface-sterilized and dehusked with 100 ml of full-strength Clorox^®^ bleach (Oakland, CA) for 2.5 h with gentle stirring and then rinsed five times with sterile distilled water [44]. The dehusked seeds were immersed in sterile distilled water in the dark at 26 °C overnight and re-sterilized with 100% bleach for another 80 min with several rounds of rinsing with sterile distilled water before being placed on callus induction medium (CIM) (Table 2). Treatment B was identical to treatment A except that the second 100% bleach sterilization step was omitted. Treatment C was similar to treatment B but the dehusked seeds were immersed in sterile distilled water for only 4 h at 26 °C before being incubated on CIM. 100 seeds were used for callus induction per replicate. As calluses grow, the induced calluses were manually broken down to pieces about 0.2 cm in size during the first 2 weeks and callus pieces were all incubated on CIM. The CIM medium was refreshed every 2 weeks. Six to eight weeks later, calluses will be ready for *Agrobacterium*-mediated genetic transformation. Callus induction rate was defined as the number of calluses induced divided by the number of seeds used.

### Regeneration of callus from parent line

Two types of regeneration medium, the MS-based and NB-based media, were tested for their ability to regenerate both solid and friable types of calluses induced by CIM according to Rengasamy et al. [44] and Li and Qu [39]. The MS-based regeneration medium used MS basal medium supplemented with vitamins, while NB-based regeneration medium used NB basal medium. Both regeneration media were supplemented with plant hormones (2 mg l^−1^ BAP + 1 mg l^−1^ IAA + 1 mg l^−1^ kinetin + 1 mg l^−1^ NAA), 500 mg l^−1^ l-proline, and 3 g l^−l^ phytagel. The calluses were incubated for 3–4 weeks with the media refreshed every 2 weeks. For rooting, the regenerated shoots (about 1–2 cm) were cut from the base of the adventitious shoots and transferred to rooting medium (RM) (Table 2).

### Vector construction and *Agrobacterium* transformation

Vector construction was modified based on the binary vector pCAMBIA1305.2, which contains a secretory *GUSPlus* gene, a kanamycin-resistant gene for bacteria selection, and a hygromycin B-resistant gene for plant selection. pCAMBIA-EV was generated by the deletion of the CaMV35S promoter and *GUSPlus* coding sequence from pCAMBIA1305.2 (2673 bp in total) performed by GenScript Corporation and confirmed by DNA sequencing (Piscataway, NJ). pCAMBIA-RFP was generated by inserting the pporRFP (*Porites porites* red fluorescent protein) expression cassette from pANIC6A [53] into pCAMBIA-EV using two restriction enzymes, *Pvu*II and *Xba*I. The pporRFP expression cassette was amplified from pANIC6a using the primer sets (pporRFP-F and pporRFP-R, Additional file 1: Table S2). The three plasmids (pCAMBIA-EV, pCAMBIA1305.2 and pCAMBIA-RFP) were introduced into *Agrobacterium* (EHA105) by a freeze–thaw method, individually [64].

### *Agrobacterium* suspension preparation

The *Agrobacterium tumefaciens* strain EHA105 harboring plasmid pTOK47 was used as the parental strain in this transformation protocol [65]. The single colony of the transformed *Agrobacterium* was inoculated into 5 ml Luria–Bertani (LB) medium with 20 mg l^−1^ rifampicin and 50 mg l^−1^ kanamycin. The culture was grown at 30 °C overnight with shaking at 200 rpm. The overnight culture was further transferred to 50 ml LB medium with the same antibiotics and grown until an OD_600_ reading of 0.6–0.8 was reached. The bacteria culture was harvested by centrifugation at 4000×*g* for 10 min, resuspended in liquid CIM medium (without plant hormones) and re-centrifuged once to wash the *Agrobacterium* culture again. The supernatant was discarded and the OD_600_ reading was adjusted to about 0.5 by suspending the pellet in an optimal amount of liquid CIM medium (without plant hormones). After adjusting the culture density, the suspension was transferred into a 100 ml sterile flask and supplemented with acetosyringone to a final concentration of 100 μM.

### *Agrobacterium*-mediated transformation of callus

The *Agrobacterium*-mediated genetic transformation was modified from Li and Qu [39]. Two days prior to the transformation, calluses were pre-cultured on fresh CIM. To start the transformation, the calluses were immersed in the prepared *Agrobacterium* suspension, with a vacuum of 0.67 atm applied for 10 min in a polypropylene vacuum desiccator (6248-15, Ace Glass Inc., Vineland, NJ) at room temperature (increase/decrease vacuum in about 0.017 atm/s for a 40 s-period), followed by agitation for 20 min at 80 rpm at 30 °C. Then, calluses were blotted on sterile tissue paper before desiccation-treatment in Petri dishes (100 mm * 15 mm, Thermo Fisher Scientific, Waltham, MA) with sterile Whatman No. 1 filter paper discs (90 mm), which were pre-wetted with 100 μl sterile water in the middle (10 calluses/plate). The desiccation treatment (co-cultivation) was performed in the dark at 26 °C for 2 days.

### Selection of transformed callus and the regeneration of transgenic plantlets

After transformation, co-cultivation and desiccation, calluses were washed with sterile water in 100 ml sterile flasks and blotted on sterile tissue paper to remove excess water. The calluses were rested on CRM for 7 days before the antibiotic selection. After resting, the calluses were subjected to three rounds of antibiotic selection (2 weeks/round) on CSM (Table 2). The first and second rounds contained 50 mg l^−1^ hygromycin B, while 100 mg l^−1^ hygromycin B was used for the final round of selection. Then, the hygromycin B-resistant calluses were transferred to REG for 3–4 weeks (Table 2) for shoot regeneration. Regenerated shoots (about ~ 1.0–1.5 cm in length) were transferred to RM medium (Table 2) for rooting.

### Genomic DNA extraction and PCR analysis

Approximately 50–100 mg of young leaves of WT or putative transgenic switchgrass plantlets (2-week-old) were collected into 2 ml safe-lock Eppendorf tubes and stored in liquid nitrogen for genomic DNA extraction. Prior to genomic DNA extraction, frozen tissue were immediately homogenized in QIAGEN TissueLyser II (85300; Qiagen Valencia, CA) under 30 Hz for 2 min. The genomic DNA was extracted using QIAGEN DNeasy Plant Mini Kit (69104; Qiagen) according to manufacturer’s instructions. The PCR analysis was performed using *Taq* 2x master mix (NEB, Ipswich, MA) with the appropriate primers, and the PCR reaction consisted of standard PCR with 35-cycles denaturing and annealing carried out at a temperature of 98 and 55 °C, respectively. The primer sets for *GUSPlus* (Gus-F and Gus-R) were derived from the literature [66], while other primer sets were designed for specific genes in each construct, which are all listed in Additional file 1: Table S2. It has been suggested the faint false-positive bands were occasionally detected in genomic DNA PCR [20, 46]; therefore, only clear, strong bands of the genomic PCR results were determined to be positive. Selection efficiency was counted as the number of genomic PCR positive switchgrass transformants divided by the regenerated hygromycin B resistant switchgrass transformants.

### Southern blot analysis

Southern blot analysis was performed according to Ramamoorthy and Kumar [44]. Genomic DNA was digested with *Hind*III restriction enzyme, fractionated on 1% (w/v) agarose gel and transferred onto a positively charged nylon membrane using TurboBlotter transfer system (GE Healthcare Bio-Sciences, Pittsburgh, PA). The blot was hybridized with digoxigenin (DIG)-labeled *hph* fragment (745 bp) as the probe, which was synthesized using PCR DIG Probe Synthesis Kit (11636090910; Roche, Indianapolis, IN) with primer set (RT_EV-F and RT_EV-R, Additional file 1: Table S2) in DIG Easy Hyb solution at 42 °C. After hybridization, the membrane was washed twice with 2× saline-sodium citrate (SSC) buffer and 0.1% SDS for 5 min, then twice with 0.5× SSC and 0.1% SDS for 15 min at 68 °C. Detection was carried out according to the manufacturer’s protocol using DIG Wash and Block Buffer Set (11636090910, Roche) and chemiluminescent substrate CDP-Star^®^ (12041677001, Roche).

### RNA isolation and reverse transcription PCR (RT-PCR)

The stem tissue of 3-month-old WT or putative transgenic switchgrass plants was frozen in liquid nitrogen and ground into fine powder using an mortar and pestle. Approximately 100 mg of stem powder was used for RNA isolation. The total RNA isolation was performed using QIAGEN RNeasy Plant Mini Kit (74904; Qiagen) according to manufacturer’s instructions. Five hundred ng of total RNA in 10 μl was reverse transcribed to cDNA in a 20 μl reaction by High Capacity cDNA Reverse Transcription Kit (4368814; Applied Biosystems, Grand Island, NY) using random hexamers according to the manufacturer’s instructions. For RT-PCR, the cDNA was diluted 20-fold and the PCR analysis was performed using Q5 High-Fidelity 2X Master Mix (M0492S; NEB) with the corresponding primers. The primer set for *GUSPlus* was the same as for genomic DNA PCR, primer sets for *Actin* and *Hph* were derived from the literature [44, 67], while primer set for *pporRFP* was designed individually, which are all listed in Additional file 1: Table S2. The PCR reaction consisted of standard PCR with 35-cycles denaturing and annealing carried out at a temperature of 98 and 55 °C, respectively. As a negative control for the genomic DNA contamination of *Agrobacterium*, 500 ng of total RNA was directly diluted 40-fold and subject to the same PCR reaction.

### Protein extraction

Total soluble proteins were isolated from 3-month-old WT or putative transgenic switchgrass plants as described previously [21]. About 1.5 g stem powder was transferred in 15 mL Falcon^®^ tubes containing 2.5 ml of cell lysis buffer [100 mM Tris–HCl (pH 6.8), 10 mM EDTA, 4 mM β-mercaptoethanol, 0.1 mM phenylmethylsulphonylfluoride (PMSF)] supplemented with cOmplete, Mini, EDTA-free Protease Inhibitor Cocktail (Roche, Indianapolis, IN) according to the manufacturer’s instructions. Protein concentrations were determined by the Bradford assay [68] using bovine serum albumin as a standard.

### Visualization of fluorescent proteins

For callus imaging, the fluorescence of pporRFP was observed using an Olympus SZX12 fluorescent stereo-dissecting microscope coupled to an Olympus U-CMAD3 digital camera and captured using SPOT imaging software (Diagnostic Instruments, Inc.; Sterling Heights, MI). The light source was an Olympus 100 W mercury lamp (Center Valley, PA). For plant tissue, the pporRFP fluorescence was visualized using a Nikon Eclipse 90i digital microscopy system coupled to a Nikon DS-Fi1 digital microscope camera (Melville, NY). The light source was a 120 W metal halide lamp in an X-Cite^®^120 Fluorescence Illuminators (EXFO, Mississauga, Ontario, Canada). A 535/30 nm excitation filter and a 600/50 nm emission filter were used for RFP imaging.

### Histochemical β-glucuronidase (GUS) staining

Histochemical GUS staining procedure was modified from the literature [69]. An amount of 52.2 mg aliquot of 5-bromo-4-chloro-3-indolyl β-d-glucuronide (X-Gluc) was dissolved in 1 ml of *N*, *N*-dimethylformamide (*N*, *N*-DMF) before adding into the GUS staining solution. Stem tissue was harvested and immediately immersed in the GUS staining solution (100 mM NaPO_4_ (pH 7.0), 1 mM potassium ferricyanide, 10 mM EDTA, 0.1% Triton X-100 and 2 mM X-Gluc). The samples (calluses or stems) were briefly vacuum-infiltrated in the GUS staining solution and kept overnight at 37 °C for staining development. Before observation, the green chlorophyll of the stem tissue was cleared with several changes of 50% ethanol and the samples were stored in 50 mM NaPO_4_ (pH 7.2) at room temperature.

## Additional file

**Additional file 1.** Supplementary information for establishing switchgrass tissue culture and *Agrobacterium*-mediated transformation protocol in this study.

## Abbreviations

BAP: 6-benzylaminopurine
CIM: callus induction medium
CRM: callus resting medium
EV: empty vector
GA_3_: gibberellic acid
GFP: green fluorescent protein
GUS: β-glucuronidase
IAA: indole-3-acetic acid
NAA: α-naphthaleneacetic Acid
REG: regeneration medium
RFP: red fluorescent protein
RM: rooting medium
SE: somatic embryogenic
