# Supplementary material for: Evaluation of parameters affecting switchgrass tissue culture: toward a consolidated procedure for Agrobacterium-mediated transformation of switchgrass (Panicum virgatum)
Source: Plant Methods. 2017 Dec 19;13:113. doi: 10.1186/s13007-017-0263-6 (PMC5735814; doi:10.1186/s13007-017-0263-6)
Supplement: Supplementary file 1 — Additional file 1. Supplementary information for establishing switchgrass tissue culture and Agrobacterium-mediated transformation protocol in this study. [file 13007_2017_263_MOESM1_ESM.pdf]

Manuscript title:

Evaluation of parameters affecting switchgrass tissue culture: toward a consolidated procedure for *Agrobacterium*-mediated transformation of switchgrass (*Panicum virgatum*)

Additional file.
File type: PDF

Contents:

- **Table S1.** Comparison of the starting materials, target genes and transformation efficiency for the reported protocols and the protocol developed and used in this study.
- **Table S2.** Primer sets used in this study.
- **Figure S1.** Seed sterilization of mature wild type (WT) switchgrass caryopses. (**A**) Switchgrass seeds were purchased from Ernst Conservation Seeds, Inc. (Meadville, PA). (Left, seeds with seed husks; right, seeds with seed coats). (**B**) Seed sterilization performed in full-strength Clorox for 2.5 h. (**C**) Sterilized seeds after several rinses with sterile distilled water to remove the peeled husks and bleach residue. (**D**) The endosperm with intact embryo should be observable after sterilization. Arrows indicate intact seeds; inset: higher magnification of the sterilized seeds in the flask.
- **Figure S2**. Comparison of seed sterilization between protocols from Li and Qu (2011) and Liu et al. (2015). (**A**) Sterilized seeds in the flask after sterilization process of Li and Qu (2011) using 100% Clorox bleach for 2.5 hour. (**B**) Sterilized seeds in the flask after sterilization process of Liu et al. (2015) using 70 % alcohol for 1 min and 50 % Clorox bleach for 2.5 hour. (**C**) Sterilized seeds before transferring to callus induction medium from (**A**); inset: higher magnification of the sterilized seeds. (**D**) Sterilized seeds before transferring to callus induction medium from (B); inset: higher magnification of the sterilized seeds.
- **Figure S3.** 100% Type II callus can be obtained by actively dividing and propagating the friable calluses. Right, higher magnification images of the Type II calluses.
- **Figure S4.** Lack of regeneration observed in samples of WT switchgrass callus raised on NB-based regeneration (REG) medium supplemented with 0.2 mg l^{-1} NAA + 1 mg l^{-1} BAP + 0.5 mg l^{-1} GA_3.
- **Figure S5.** Impact of hygromycin B concentration on WT rooting (killing curve).
- **Figure S6.** Colony PCR results from the *Agrobacteria* transformants with different vectors (pCAMBIA-EV, pCAMBIA-RFP, and pCAMBIA-1305.2). Primer set 1 (EV-F, EV-R) is specific for *Hph* gene. Primer set 2 (RFP-F, RFP-R) is for *pporRFP* gene. Primer set 3 (Gus-F, Gus-R) is for *GUSPlus* gene.

- **Figure S7.** Vacuum-infiltration and desiccation treatment procedure for *Agrobacteria*-meditated callus transformation. (**A**) Transferred 2-day pre-culture WT calluses to the prepared *Agrobacterium* suspension. (**B**) Vacuum-infiltration the *Agrobacterium* suspension in plastic desiccator. (**C**) Desiccation treatment using 10 calluses with 100 μl sterile water in the middle. (**D**) Two days after desiccation treatment.
- **Figure S8.** The confirmation of the transgene using 3-month-old greenhouse-grown transgenic switchgrass plants by genomic DNA PCR. Primer set A (EV-F, EV-R) is specific for *Hph* gene. Primer set B (RFP-F, RFP-R) is for *pporRFP* gene. Primer set C (Gus-F, Gus-R) is for *GUSPlus* gene.
- **Figure S9.** Determination of genomic DNA amount for Southern blot analysis by chemiluminescent detection method using *Hph* DIG-labeled probe of pCAMBIA-1305.2 transgenic. NC, negative control; P, positive control.
- **Figure S10.** Detection of transgene gene expression using reverse transcription PCR (RT-PCR). (**A**) pCAMBIA-EV transformed transgenic switchgrass. (**B**) pCAMBIA-RFP transformed transgenic switchgrass. (**C**) *GUSPlus* gene in pCAMBIA-1305.2 transformed transgenic switchgrass. Lane 1, RNA; lane 2, PCR using cDNA as template by gene-specific primer sets for *Hph* gene (RT_EV-F + RT_EV-R in **A**), *pporRFP* gene (RT_RFP-F and RT_RFP-R in **B**) and *GUSPlus* (Gus-F + Gus-R in **C**); lane 3, PCR using RNA as template by gene-specific primer sets for *Hph* gene (RT_EV-F + RT_EV-R in **A**), *pporRFP* gene (RT_RFP-F and RT_RFP-R in **B**) and *GUSPlus* (Gus-F + Gus-R in **C**); lane 4, PCR RT-PCR using primer sets for *ACTIN* gene (RT_ACTIN2-F + RT_ACTIN2-R).
- **Figure S11.** Detection of pporRFP expression in root tissue of empty vector (EV) and pCAMBIA-RFP (RFP) transformed switchgrass. (**A**) Bright field image. (**B**) Epi-fluorescence image under red fluorescence filter.
- **Figure S12.** Detection of the pporRFP fluorescence in the total stem protein extract of pCAMBIA-RFP switchgrass transgenic lines. (**A**) Bright field image. (**B**) Epi-fluorescence image. Each droplet has 2μg of total stem protein extract.

Table S1. Comparison of the starting materials, target genes and transformation efficiency for the reported protocols and the protocol developed and used in this study.

	Cultivar	*Agrobacterium* Strain	Callus Origin	Callus Type	Gene	Selection Marker	Transformation Efficiency (%)	Reference
1	Alamo	AGL1	Somatic embryos*<br>Embryogenic calluses<br>Mature caryopses<br>Basal part of plantlets	N/A	*GUS*	*Bar*	3.4#	Somleva et al., 2002; 2008
2	Alamo 2	EHA105	Inflorescences	Type II	*pporRFP*	*Hph*	4.4#	Burris et al., 2009
3	Alamo	EHA105*<br>AGL1*<br>LBA4404	Mature caryopses<br>Inflorescence<br>Nodes	N/A	*GUS*	*Hph*	N/A	Xi et al., 2009
4	Alamo<br>Performer<br>Colony	EHA105	Mature caryopses	Type II	*GFP*	*Hph*	Alamo (56.6)<br>Colony (44.6)<br>Performer (91.6)	Li and Qu, 2011
5	Alamo*<br>Cave-in-Rock<br>(CIR)	EHA105*<br>LBA4404<br>GV3101	Seedlings<br>(basal part)	N/A	*GUS*	*Bar**<br>*Hph**<br>*Npt*	Alamo (4.9-5.9)<br>CIR (0.0)	Song et al., 2012
6	Alamo	AGL1	Mature caryopses	Type II	*GFP*	*Hph*	6.0	Ramamoorthy and Kumar, 2012
7	Alamo	EHA101	Mature caryopses	Type I	*GFP*<br>*GUS*	*Bar*	2.0 -17.4	Ogawa et al., 2014
8	Alamo<br>Performer<br>Blackwell<br>Dacotah	EHA105	Mature caryopses	Type II	*GUS*	*Hph*	Alamo (72.8)<br>Dacotah (8.0)	Liu et al., 2015
9	Alamo<br>Kanlow<br>Blackwell<br>CIR<br>Trailblazer	EHA101	Mature caryopses	Type I	*GFP*<br>*GUS*	*Bar*	Alamo (12.5-59.2)<br>Kanlow (6.3- 20.0)<br>Trailblazer (7.5-16.3)	Ogawa et al., 2016
10	Alamo	EHA105	Mature caryopses	Type I<br>Type II	*pporRFP*<br>*GUS*	*Hph*	50.0-100.0^	This study

GUS, β-glucuronidase; *pporRFP*, red fluorescence protein; *GFP*, green fluorescence protein

Bar, phosphinothricin acetyltransferase; *Hph*, hygromycin phosphotransferase; *Npt*, neomycin phosphotransferase

N/A, not applicable; *, more effective; #, values are according to Ogawa et al., 2014; ^, selection efficiency

Table S2. Primer sets used in this study.

Primer	Sequence (5' to 3')	Target	Amplicon (bp)	Reference
pporRFP-F	ATCG**CAGCTG**CCACTGGAGAGGGGCACA	pporRFP expression cassette (pANIC6A)	3, 014	This study
pporRFP-R	CGATTCTAGAGTTTAATTCCCGATCTAG			
EV-F	CGTTATGTTTATCGGCACTTTGCAT	*Hygromycin B phosphotransferase* (*Hph*) (pCAMBIA-EV)	950	This study
EV-R	AGCGAAACCCTATAGGAACCCTAAT			
RFP-F	GCCTTTGCCATTTTCTATTGACATT	*pporRFP* (pCAMBIA-RFP)	836	This study
RFP-R	GTTTAATTCCCGATCTAG			
Gus-F	CAAGCACCGAGGGCCTGAGC	*GUSPlus* (pCAMBIA1305.2)	574	Muthukumar et al., 2013
Gus-R	CTCAGTCGCCGCCTCGTTGG			
RT_EV-F	CAACCAAGCTCTGATAGAGT	*Hph* (pCAMBIA-EV)	745	Ramamoorthy and Kumar 2012
RT_EV-R	GAAGAATCTCGTGCTTTCA			
RT_RFP-F	Same as RFP-F	*pporRFP* (pCAMBIA-RFP)	549	This study
RT_RFP-R	GAAGAATCTCGTGCTTTCA			
RT_ACTIN2-F	GCGAGCTTCCCTGTAGGTA	*Pv_ACTIN2* (FL724919.1)	317	Xu et al., 2011
RT_ACTIN2-R	CACACGGAGCTCGTTGTAGA			

***Pvu*II** site in bold and ***Xba*I** site underlined

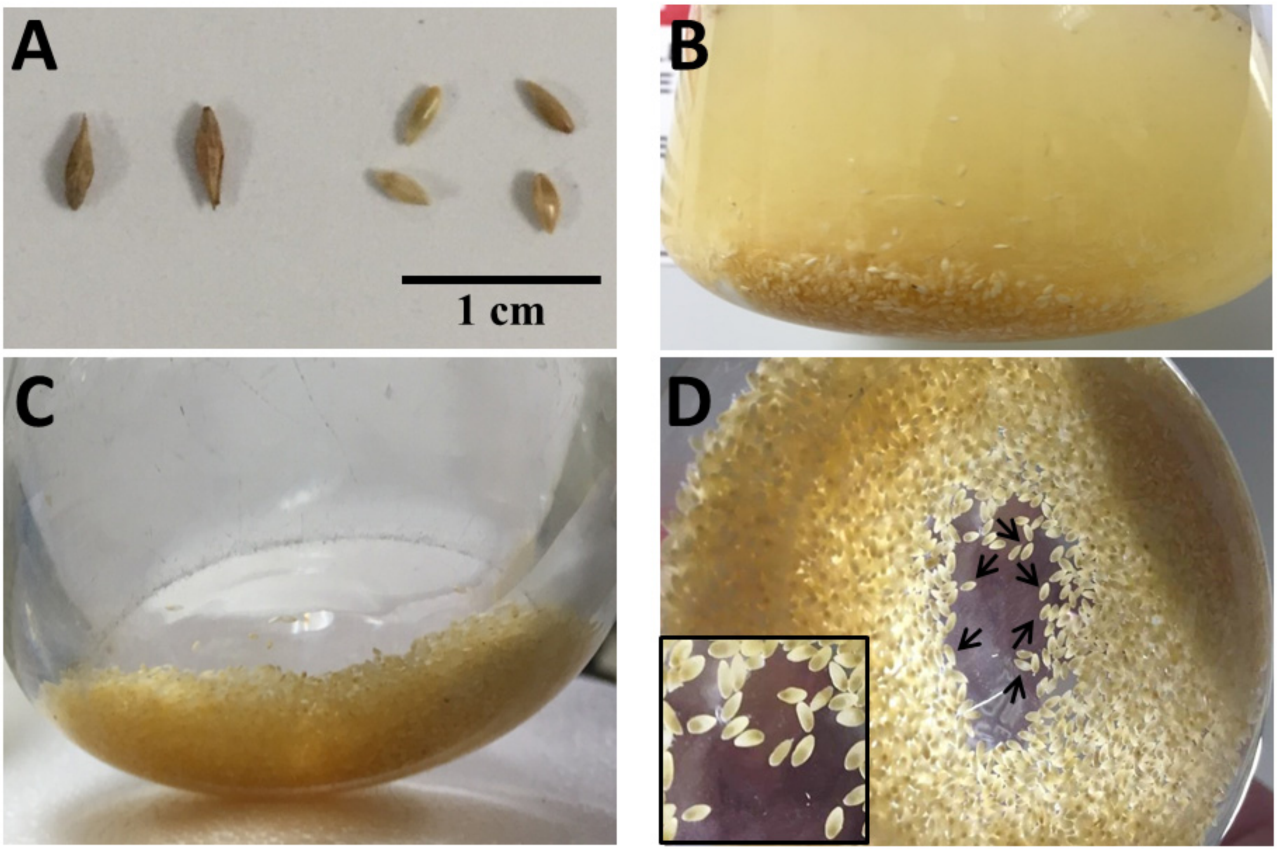

Figure S1. Seed sterilization of mature wild type (WT) switchgrass caryopses. (**A**) Switchgrass seeds were purchased from Ernst Conservation Seeds, Inc. (Meadville, PA). (Left, seeds with seed husks; right, seeds with seed coats). (**B**) Seed sterilization performed in full-strength Clorox for 2.5 h. (**C**) Sterilized seeds after several rinses with sterile distilled water to remove the peeled husks and bleach residue. (**D**) The endosperm with intact embryo should be observable after sterilization. Arrows indicate intact seeds; inset: higher magnification of the sterilized seeds in the flask.

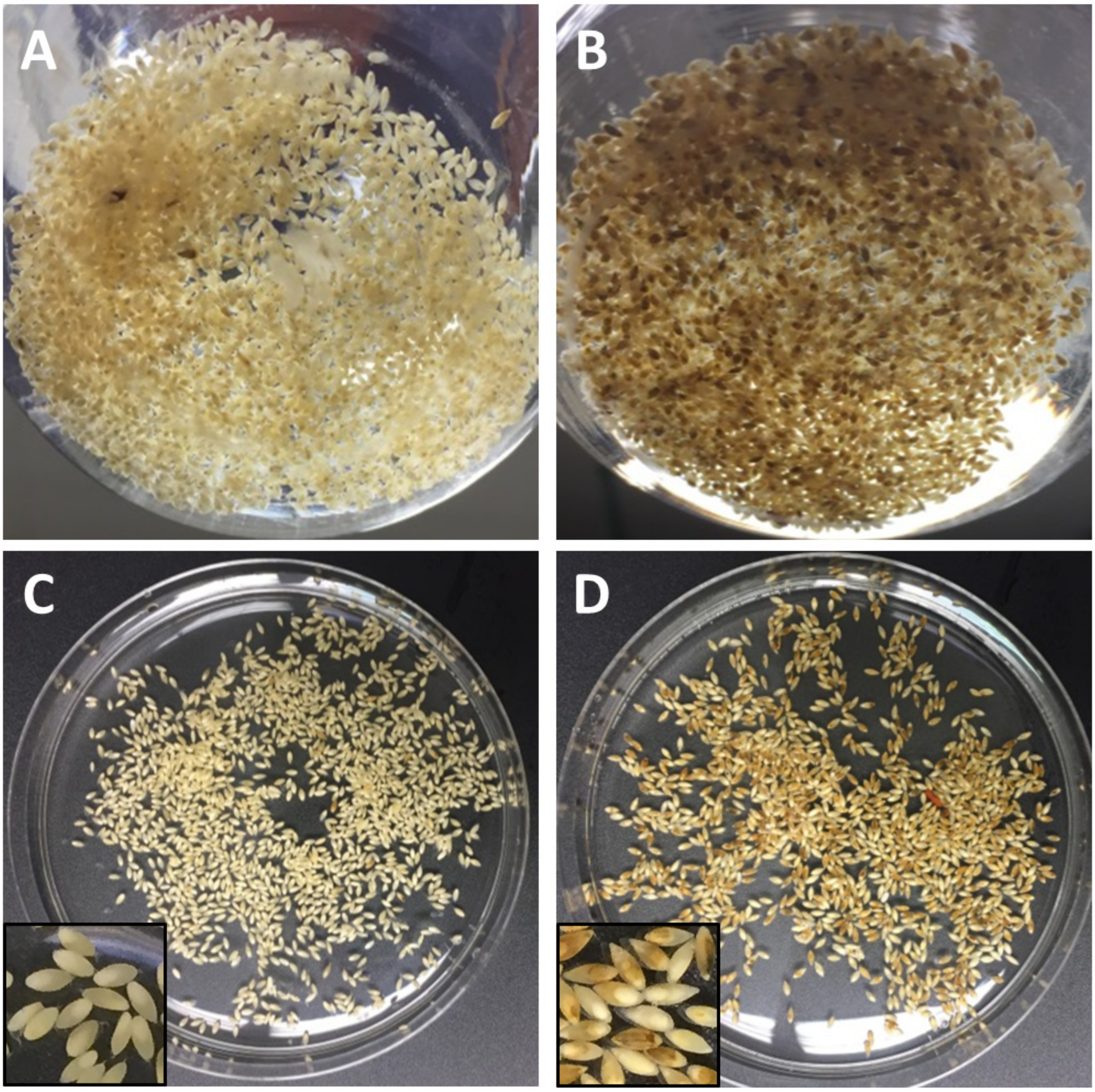

Figure S2. Comparison of seed sterilization between protocols from Li and Qu (2011) and Liu et al. (2015). (**A**) Sterilized seeds in the flask after sterilization process of Li and Qu (2011) using 100% Clorox bleach for 2.5 hour. (**B**) Sterilized seeds in the flask after sterilization process of Liu et al. (2015) using 70 % alcohol for 1 min and 50 % Clorox bleach for 2.5 hour. (**C**) Sterilized seeds before transferring to callus induction medium from (**A**); inset: higher magnification of the sterilized seeds. (**D**) Sterilized seeds before transferring to callus induction medium from (B); inset: higher magnification of the sterilized seeds.

.

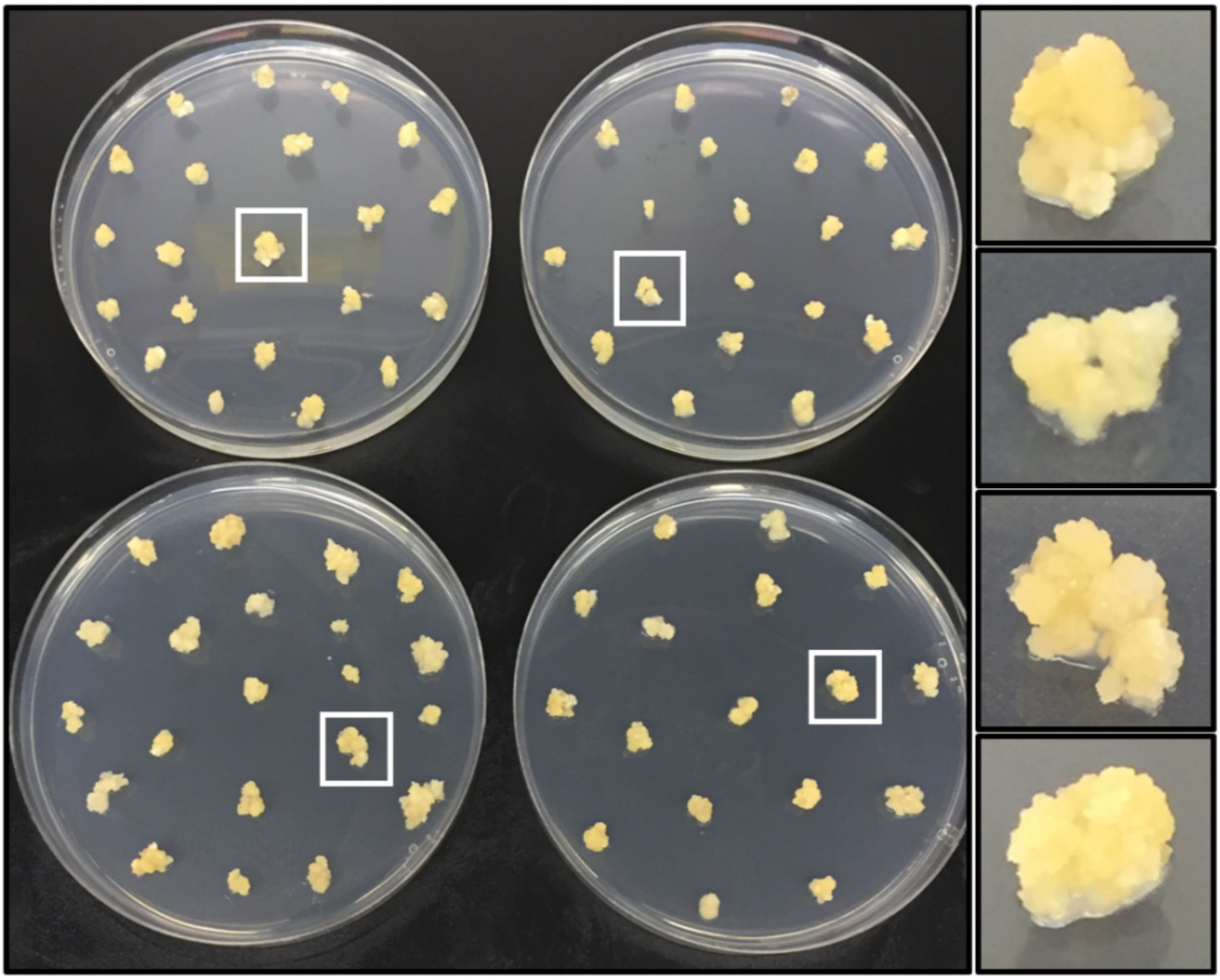

Figure S3. 100% of Type II callus can be obtained by actively dividing and propagating the friable calluses. Right, higher magnification images of the Type II calluses.

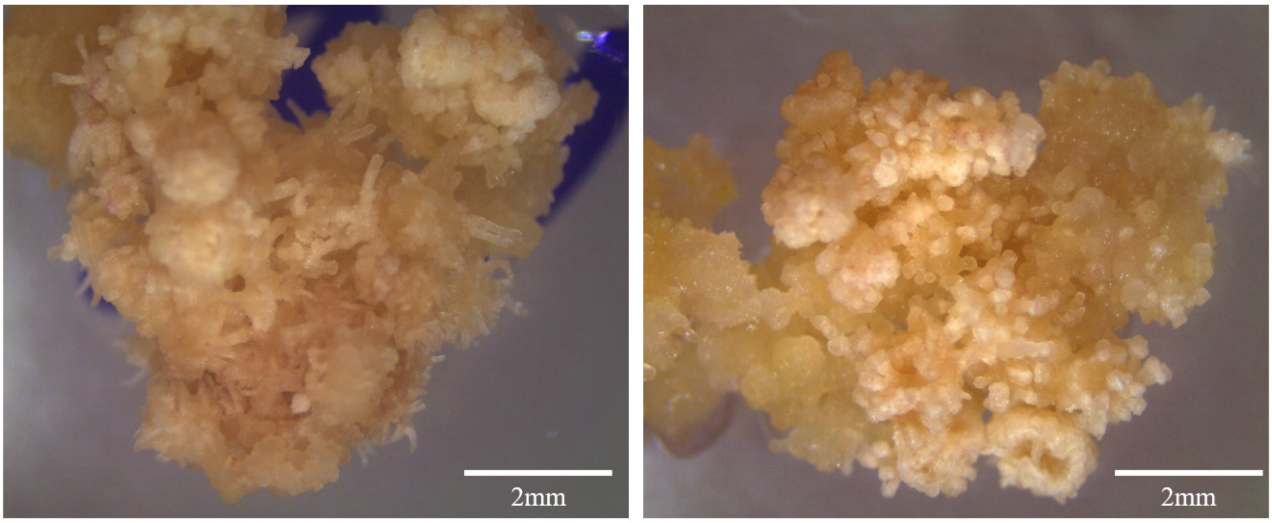

Figure S4. Lack of regeneration observed in samples of WT switchgrass callus raised on NB-based regeneration (REG) medium supplemented with 0.2 mg l^{-1} NAA + 1 mg l^{-1} BAP + 0.5 mg l^{-1} GA_3.

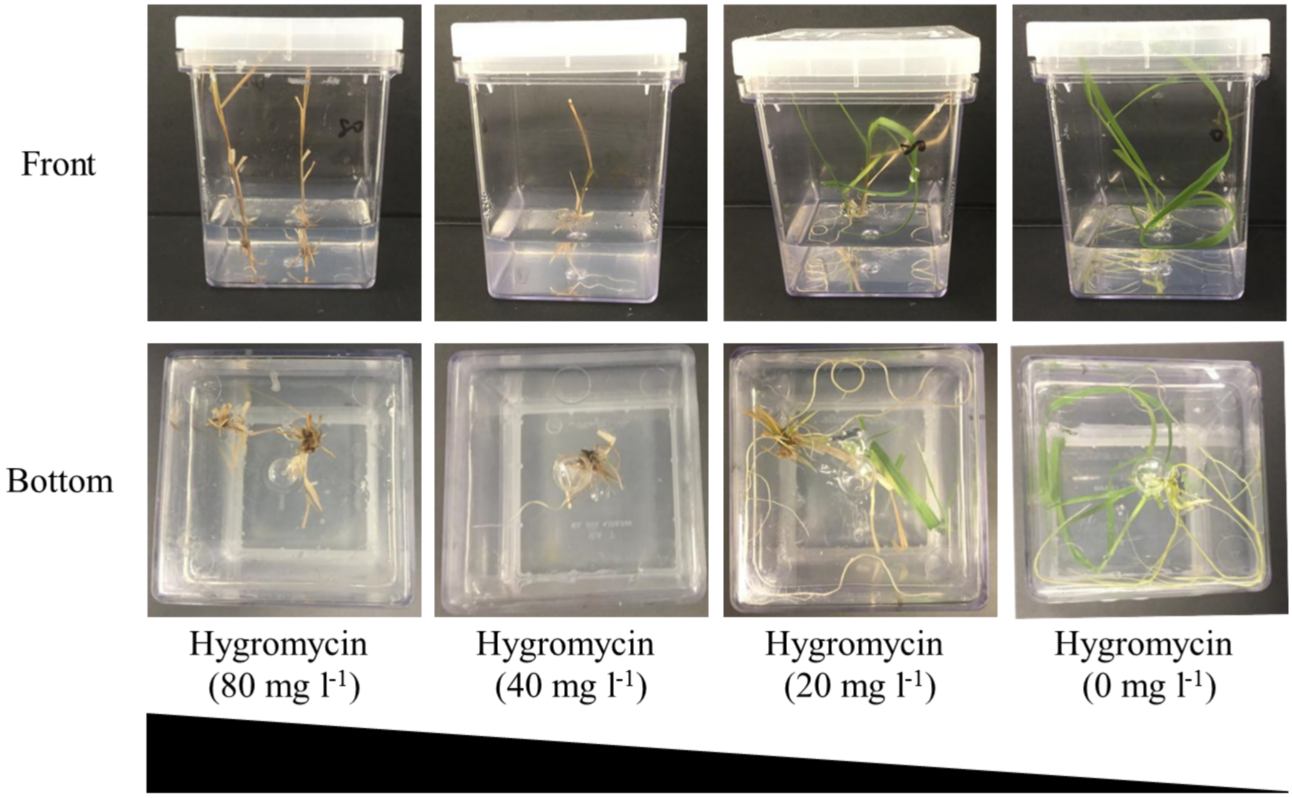

Figure S5. Impact of hygromycin B concentration on WT rooting (killing curve). From left to right, the concentration is reduced from 80 mg l^{-1} to 0 mgl^{-1} in the RM. Rooting was drastically reduced when hygromycin B concentration reached 40 mg l^{-1} or above in the RM and the growth of WT switchgrass was totally inhibited at 80 mg l^{-1} hygromycin B.

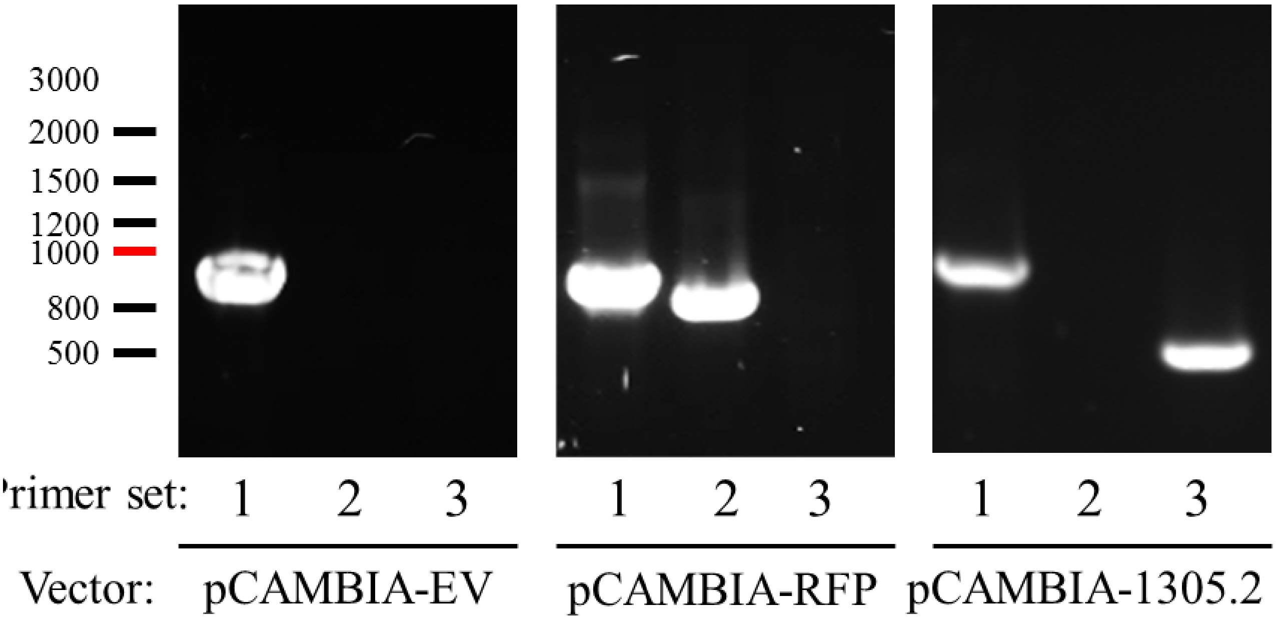

Figure S6. Colony PCR results from the *Agrobacteria* transformants with different vectors (pCAMBIA-EV, pCAMBIA-RFP, and pCAMBIA-1305.2). Primer set 1 (EV-F, EV-R) is specific for *Hph* gene. Primer set 2 (RFP-F, RFP-R) is for *pporRFP* gene. Primer set 3 (Gus-F, Gus-R) is for *GUSPlus* gene.

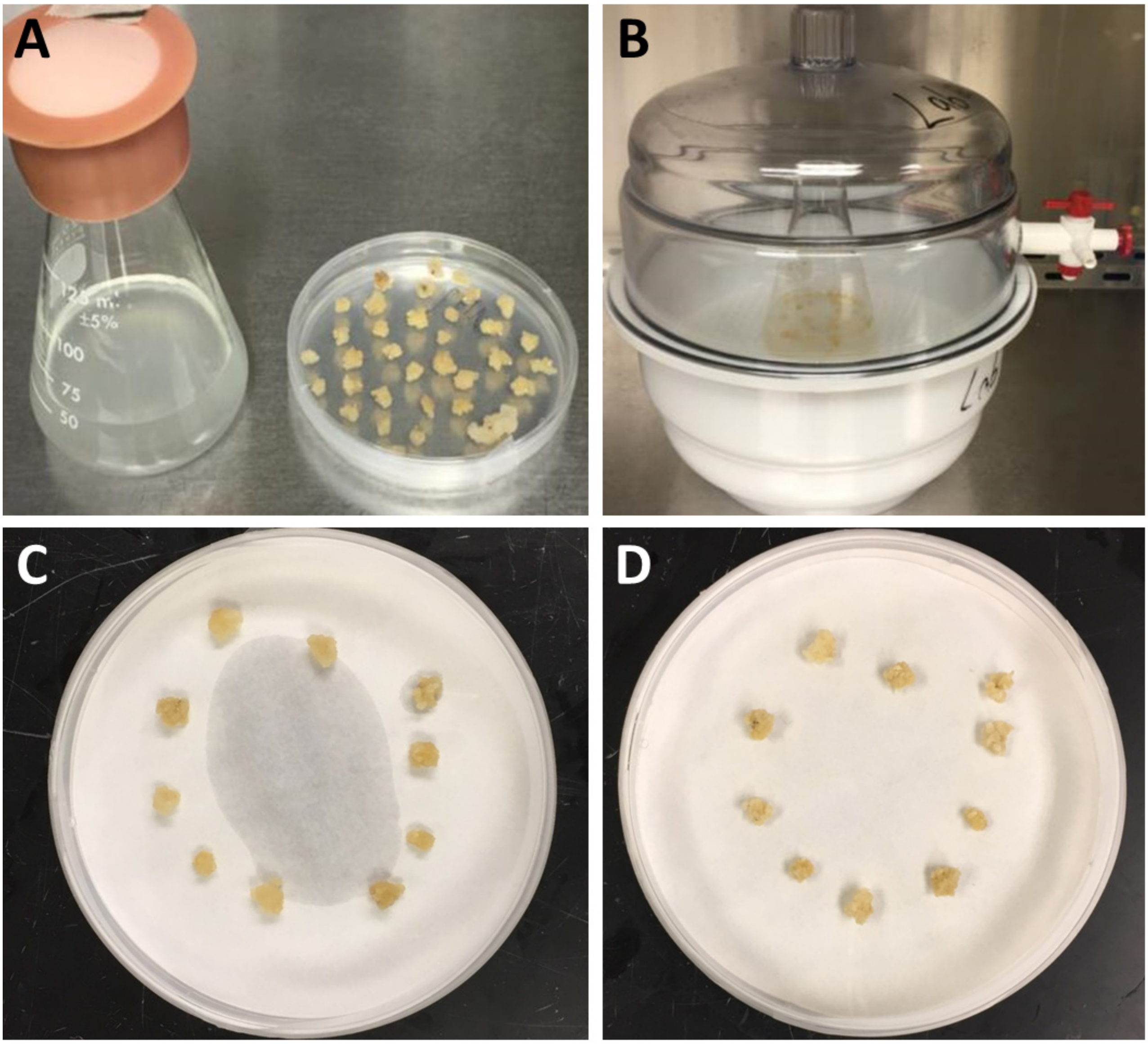

Figure S7. Vacuum-infiltration and desiccation treatment procedure for *Agrobacteria*-meditated callus transformation. (**A**) Transferred 2-day pre-culture WT calluses to the prepared *Agrobacterium* suspension. (**B**) Vacuum-infiltration the *Agrobacterium* suspension in plastic desiccator. (**C**) Desiccation treatment using 10 calluses with 100 μl sterile water in the middle. (**D**) Two days after desiccation treatment.

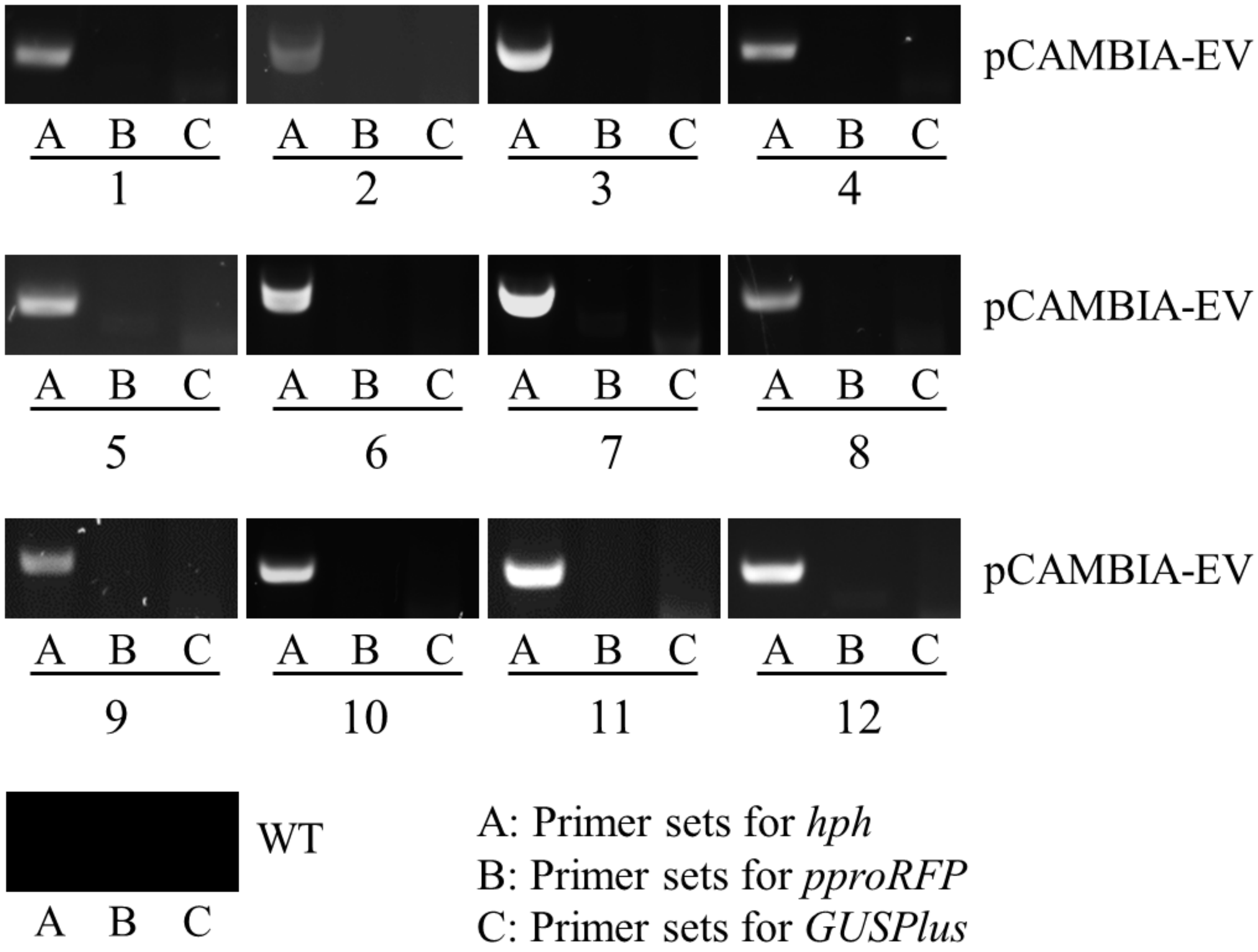

Figure S8. The confirmation of the transgene using 3-month-old greenhouse-grown transgenic switchgrass plants by genomic DNA PCR. Primer set A (EV-F, EV-R) is specific for *Hph* gene. Primer set B (RFP-F, RFP-R) is for *pporRFP* gene. Primer set C (Gus-F, Gus-R) is for *GUSPlus* gene.

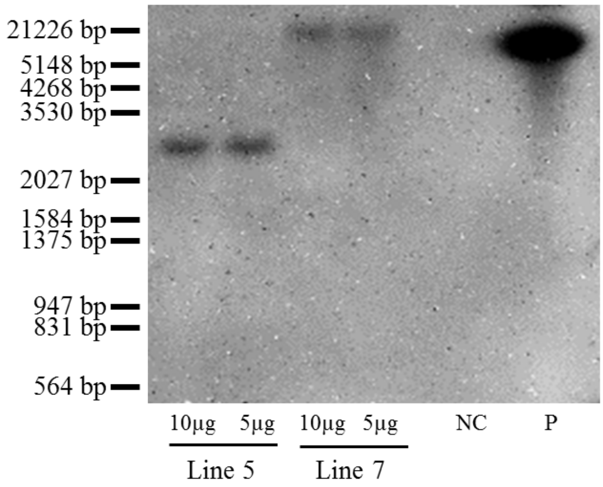

Figure S9. Determination of genomic DNA amount for Southern blot analysis by chemiluminescent detection method using *Hph* DIG-labeled probe of pCAMBIA-1305.2 transgenic. NC, negative control; P, positive control.

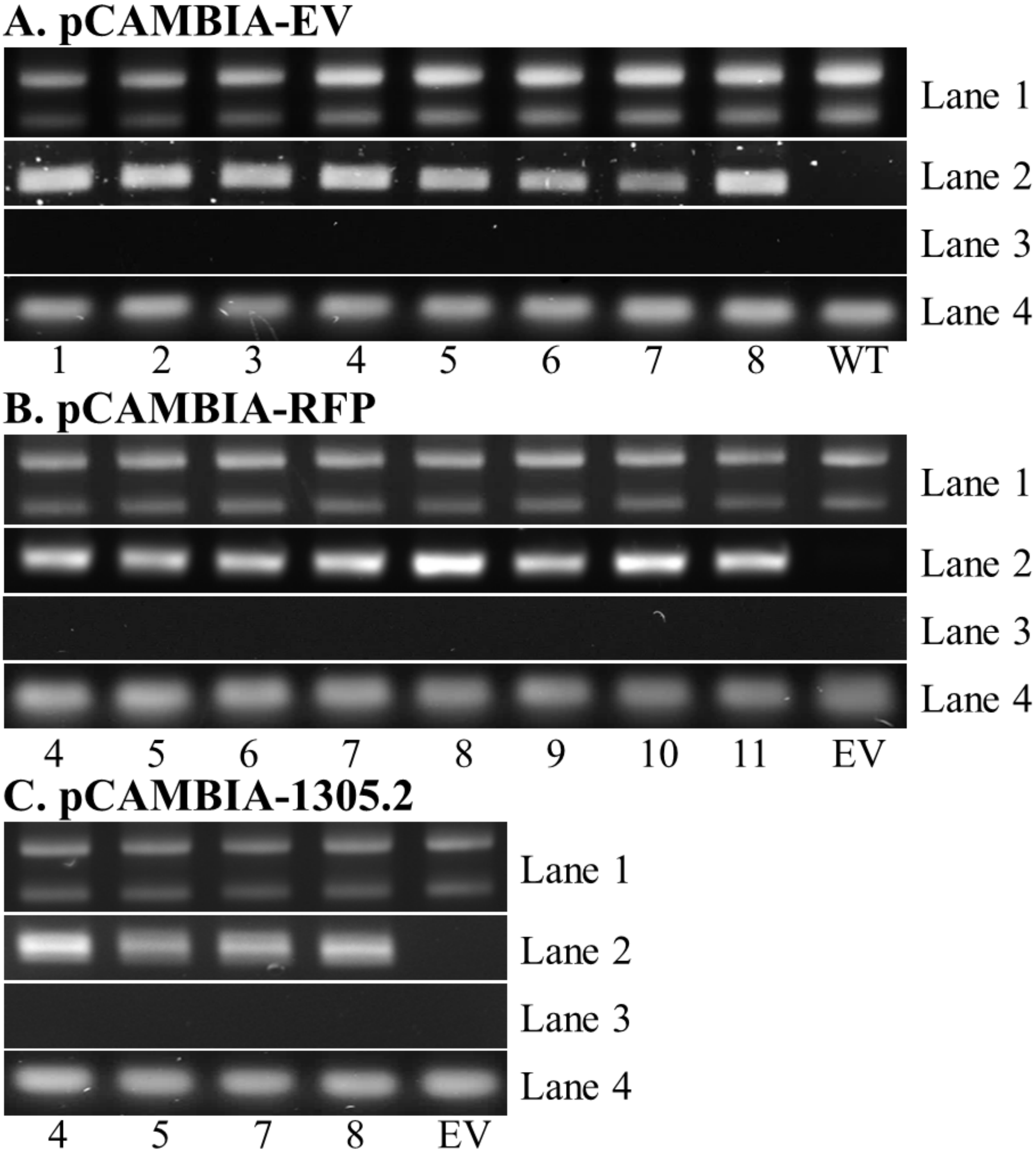

Figure S10. Detection of transgene gene expression using reverse transcription PCR (RT-PCR). (**A**) pCAMBIA-EV transformed transgenic switchgrass. (**B**) pCAMBIA-RFP transformed transgenic switchgrass. (**C**) *GUSPlus* gene in pCAMBIA-1305.2 transformed transgenic switchgrass. Lane 1, RNA; lane 2, PCR using cDNA as template by gene-specific primer sets for *Hph* gene (RT_EV-F + RT_EV-R in **A**), *pporRFP* gene (RT_RFP-F and RT_RFP-R in **B**) and *GUSPlus* (Gus-F + Gus-R in **C**); lane 3, PCR using RNA as template by gene-specific primer sets for *Hph* gene (RT_EV-F + RT_EV-R in **A**), *pporRFP* gene (RT_RFP-F and RT_RFP-R in **B**) and *GUSPlus* (Gus-F + Gus-R in **C**); lane 4, PCR RT-PCR using primer sets for *ACTIN* gene (RT_ACTIN2-F + RT_ACTIN2-R).

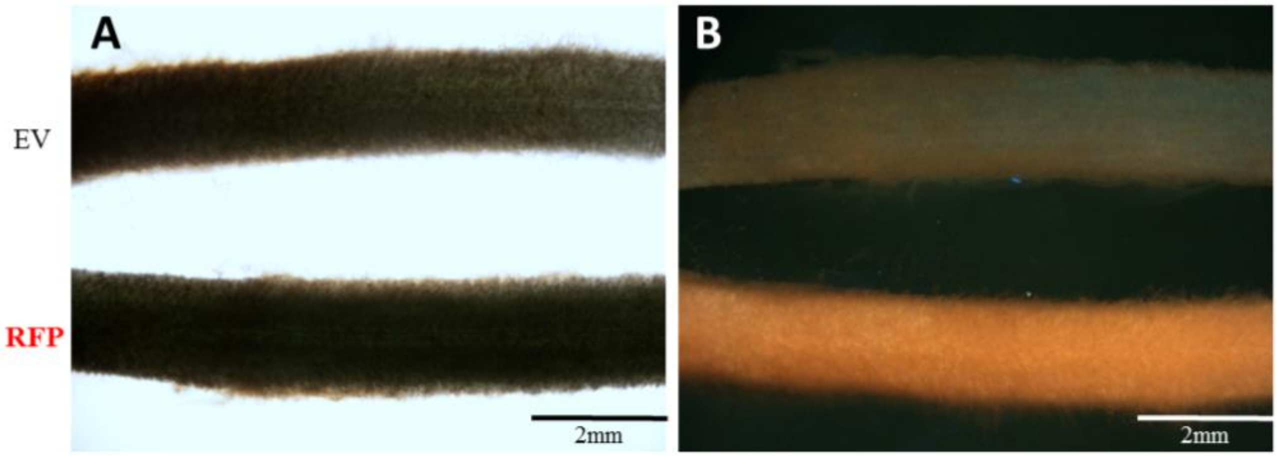

Figure S11. Detection of pporRFP expression in root tissue of empty vector (EV) and pCAMBIA-RFP (RFP) transformed switchgrass. (**A**) Bright field image. (**B**) Epi-fluorescence image under red fluorescence filter.

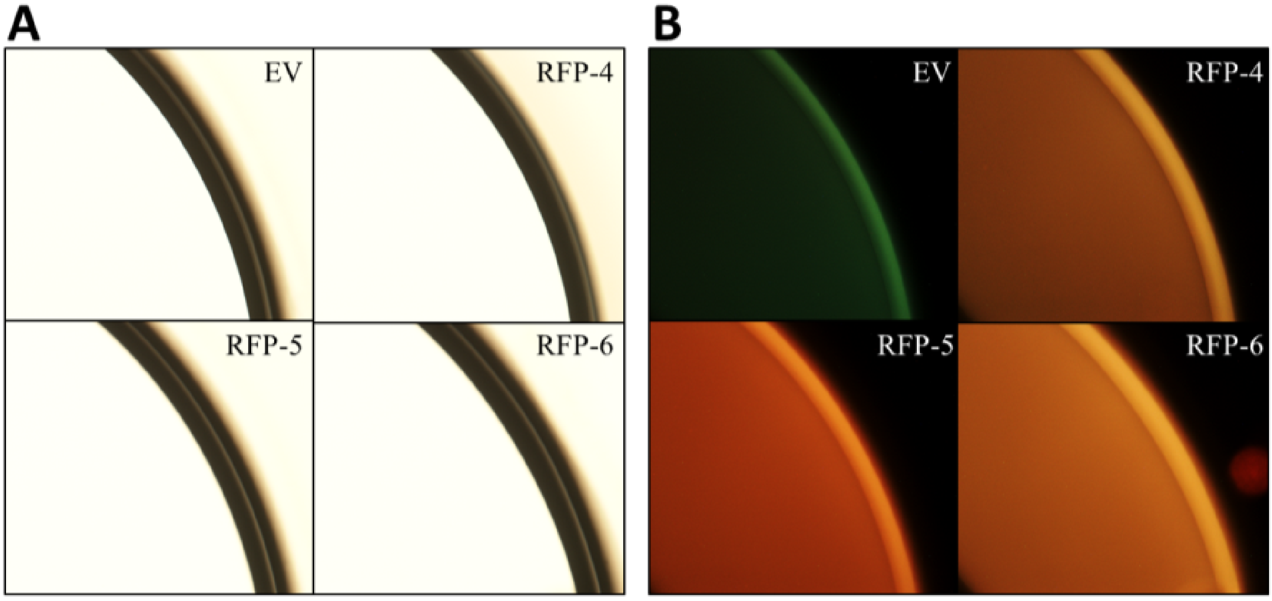

Figure S12. Detection of the pporRFP fluorescence in the total stem protein extract of pCAMBIA-RFP switchgrass transgenic lines. (**A**) Bright field image. (**B**) Epi-fluorescence image under red fluorescence filter. Each droplet has 2μg of total stem protein extract.
